# Transcranial Direct Current Stimulation Modulates Neuronal Activity and Learning in Pilot Training

**DOI:** 10.3389/fnhum.2016.00034

**Published:** 2016-02-09

**Authors:** Jaehoon Choe, Brian A. Coffman, Dylan T. Bergstedt, Matthias D. Ziegler, Matthew E. Phillips

**Affiliations:** ^1^HRL Laboratories LLCMalibu, CA, USA; ^2^Department of Psychiatry, The University of PittsburghPittsburgh, PA, USA; ^3^Psychology Clinical Neuroscience Center, The University of New MexicoAlbuquerque, NM, USA; ^4^Department of Sports Medicine, Pepperdine UniversityMalibu, CA, USA; ^5^Advanced Technologies Laboratories, Lockheed MartinArlington, VA, USA

**Keywords:** tDCS, EEG, fNIRS, DLPFC, M1, flight simulation, skill learning

## Abstract

Skill acquisition requires distributed learning both within (online) and across (offline) days to consolidate experiences into newly learned abilities. In particular, piloting an aircraft requires skills developed from extensive training and practice. Here, we tested the hypothesis that transcranial direct current stimulation (tDCS) can modulate neuronal function to improve skill learning and performance during flight simulator training of aircraft landing procedures. Thirty-two right-handed participants consented to participate in four consecutive daily sessions of flight simulation training and received sham or anodal high-definition-tDCS to the right dorsolateral prefrontal cortex (DLPFC) or left motor cortex (M1) in a randomized, double-blind experiment. Continuous electroencephalography (EEG) and functional near infrared spectroscopy (fNIRS) were collected during flight simulation, n-back working memory, and resting-state assessments. tDCS of the right DLPFC increased midline-frontal theta-band activity in flight and n-back working memory training, confirming tDCS-related modulation of brain processes involved in executive function. This modulation corresponded to a significantly different online and offline learning rates for working memory accuracy and decreased inter-subject behavioral variability in flight and n-back tasks in the DLPFC stimulation group. Additionally, tDCS of left M1 increased parietal alpha power during flight tasks and tDCS to the right DLPFC increased midline frontal theta-band power during n-back and flight tasks. These results demonstrate a modulation of group variance in skill acquisition through an increasing in learned skill consistency in cognitive and real-world tasks with tDCS. Further, tDCS performance improvements corresponded to changes in electrophysiological and blood-oxygenation activity of the DLPFC and motor cortices, providing a stronger link between modulated neuronal function and behavior.

## Introduction

There has recently been a rapid increase in the number of published studies in the field of neuromodulation due to the availability of non-invasive stimulation technologies such as transcranial direct current stimulation (tDCS). New tools for training enhancement are emerging which target specific, basic cognitive functions, with the goal of increasing performance in high-level, real-world tasks, such as pilot training. For example, Clark et al. (2012) demonstrated enhanced concealed image detection training with tDCS. Others have observed enhanced skill learning with tDCS in spatial and verbal working memories (Martin et al., 2014; Richmond et al., 2014), language acquisition (Flöel et al., 2008) and motor skills development (Banissy and Muggleton, 2013; Reis et al., 2015; Rumpf et al., 2015). For a review of tDCS enhancements (see Coffman et al., 2014).

Computerized cognitive training methods have been only moderately successful in enhancing performance (Ball et al., 2002). However, computerized procedural training (flight simulation) has been an important part of airplane pilot training since the mid 1970's. Commercial and military pilot training programs now utilize flight simulation extensively for training basic flight and combat skills (Bell and Waag, 1998; Rosenkopf and Tushman, 1998). Research on the effectiveness of flight simulator training has historically been limited by the high cost of full flight simulators, and occurs in the context of ongoing pilot training programs, rather than unbiased third-party research programs (Hays et al., 1992; Rosenkopf and Tushman, 1998). The field has recently overcome this limitation by the commercialization of relatively low-cost flight simulator devices available for purchase and use in standard research environments. These personal computer-based flight simulators are also used in various contexts for flight training (Koonce and Bramble, 1998), lending ecological validity to simulator studies.

Piloting an airplane is a demanding task requiring skillful execution of learned procedures. This has been observed as a correlation between flight simulator performance and measures of reasoning and working memory in general aviation pilots (Causse et al., 2011), and a concurrent decline in working memory and flight errors (Dismukes, 2008; Engle, 2010). Furthermore, neurophysiological markers of both short-term (e.g., fatigue) and long-term (e.g., expertise) cognitive functions correlate with behavioral performance (Ayaz et al., 2013; Borghini et al., 2014). Pilot skill development requires a synthesis of multiple cognitive faculties, many of which are enhanced by tDCS and include: dexterity (Boggio et al., 2006), mental arithmetic (Hauser et al., 2013), cognitive flexibility (Chrysikou et al., 2013), visuo-spatial reasoning (Heimrath et al., 2012), and working memory (Gill and Hamilton, 2014)—an important predictor of flight situation awareness in novices (Sohn and Doane, 2004).

Working memory is linked primarily with brain activity in the dorsolateral prefrontal cortex (DLPFC) (Courtney et al., 1996; Braver et al., 1997; Curtis and D'Esposito, 2003), an area often targeted by non-invasive brain stimulation in cognitive research. Most researchers agree that tDCS of DLPFC has substantial effects on working memory (for a review see Coffman et al., 2014); however, Horvath et al. (2015) recently reported disconfirming evidence for this hypothesis in a meta-analysis of selected studies investigating the cognitive effects of tDCS. In this meta-analysis, tDCS did not have a significant effect on any cognitive measure. However, their approach may be confounded by calculation of effect sizes based only on post-stimulation scores, rather than accounting for pre-stimulation differences between groups. Chhatbar and Feng (2015) illustrated this issue in their response paper, where they show substantial effects of tDCS when calculating effect sizes from pre-post difference scores rather than post-stimulation scores alone.

The focality of stimulation is also a critical component of tDCS-driven behavioral changes, and this aspect of experimental design is difficult to capture in meta-study. Large pad-type electrodes used in previous studies have comparatively poor focality and target current intensity as compared to the multiple electrode montage approach (Dmochowski et al., 2011). Finite elements modeling work with MRI-derived brain models performed by various groups demonstrate optimization of currents to the brain that improve focality and intensity to areas of interest by 80 and 98%, respectively (Bikson et al., 2009; Datta et al., 2011, 2012; Dmochowski et al., 2011; Faria et al., 2011; Edwards et al., 2013). The importance of this modeling work is underscored by clinical investigations that show differences in targeting and stimulation intensity results in marked differences in behavioral output and stimulation efficacy (Valle et al., 2009; Moliadze et al., 2010; Mendonca et al., 2011). Finally, Santarnecchi et al. (2015) have suggested that the impact of tDCS on target brain structures is dependent on not only the placement of electrodes and current density, but also the current state of activity in those brain areas. This crucial point is often overlooked in tDCS research, and investigators should carefully consider the cognitive task performed during stimulation to maximize the desired effect.

Despite recent controversy over the effects of tDCS on working memory, tDCS applied to specific brain regions has been reported to improve behavioral performance in a diverse array of cognitive categories: attention (Coffman et al., 2014), reaction time (Teo et al., 2011), object recognition (Clark et al., 2012), memory (Manenti et al., 2013), creativity (Chrysikou et al., 2013), and motor skill acquisition (Nitsche et al., 2003). In addition to acute improvement of various performance measures, some laboratories have also observed persistence of cognitive enhancement even after the electrical current is removed (Snowball et al., 2013; Lefebvre et al., 2014). These results indicate that, in some cases, stimulation need only be applied initially or periodically to achieve continual performance gains. Although the modulation of procedural learning through enhancement of working memory has remained an open question in the field, non-invasive brain stimulation methods are potential vehicles to enhance learning and performance and nootropic benefits for commercial and military applications (Clark et al., 2012; Phillips and Ziegler, 2014).

The application of neuroimaging techniques, such as functional near-infrared spectroscopy (fNIRS) and electroencephalography (EEG), allow the precise measurement of spatial and dynamic functional brain activity. The development of these non-invasive, low overhead and high-resolution tools have given investigators the ability to observe the activity of the human brain *in vivo* with an unprecedented degree of control (Been et al., 2007; McKendrick et al., 2015).

EEG results confirm tDCS-related modulation of brain processes involved in working memory, as evidenced by increased midline frontal theta-band oscillatory brain activity (MFT) during a working memory task (Miller et al., 2015). MFT is most commonly measured during maintenance of information in working memory, and reflects theta coupling between the DLPFC and anterior cingulate cortex (Sauseng et al., 2004). MFT is positively correlated with attentional demands during mental calculation (Ishii et al., 2014) and working memory load (Jensen and Tesche, 2002), and theta-band synchrony between frontal and parietal areas is directly related to individual working memory capacity (Palva et al., 2010). Further evidence supporting the functional relationship comes from studies temporarily disrupting the DLPFC with transcranial magnetic stimulation—leading to performance decrements in working memory tasks (Grafman et al., 1994; Pascual-Leone and Hallett, 1994). Other frequency bands have also been implicated in working memory and attentional control. For example, tonic increases (and phasic decreases) in parietal alpha-band power reflects greater perceptual involvement for tasks requiring attention to the environment (Klimesch, 1999), suggesting a role of alpha in perception. Furthermore, Sauseng et al. (2009) showed that alpha band activity over sensorimotor areas indicates greater excitability in that region, as measured with transcranial magnetic stimulation. Therefore, stimulation of either M1 or DLPFC could increase tonic alpha band activity in this study compared to sham by enhancing sensorimotor excitability and/or perceptual involvement.

Other imaging studies, employing fNIRS have found significant correlations between cognitive performance and blood oxygenation in the DLPFC (Yanagisawa et al., 2010; McKendrick et al., 2014). fNIRS is an non-invasive imaging technique that measures the relative concentrations of oxygenated (Hboxy) and deoxygenated (Hbdeoxy) hemoglobin to infer neuronal activity. fNIRS relies on differences in the near infrared absorption spectra of oxygenated and deoxygenated hemoglobin along with a neuro-vascular hemodynamic response function to relate relative chances in localized cerebral blood flow to neuronal activity (Villringer et al., 1993).

Hbdeoxy and total hemoglobin concentrations (Hbtot) are linked to levels of cognitive workload in the anterior prefrontal cortex (PFC) (Ayaz et al., 2012). For example, using a Scarborough adaptation of the Tower of London task, Ruocco et al. (2014) found that difficult problems were associated with greater Hboxy concentrations in the DLPFC relative to a baseline condition. The study also found that participants who scored higher in deliberation, or careful thinking, before acting, showed greater activation in this same region, regardless of task difficulty. The magnitude of Hbtot and Hbdeoxy concentration changes in specific brain regions has been used as a proxy for mental workload and expertise. Hbtot levels increase in the PFC during difficult trials in the N-back task, suggesting greater recruitment of neural resources (Herff et al., 2013). In addition, during a complex flight task, Hbtot levels decrease in the PFC over a 9-day learning period with progression from beginner to intermediate and finally advanced levels of performance (Ayaz et al., 2012). Furthermore, blood oxygenation level-dependent (BOLD) responses, which correlate with Hboxy, Hbdeoxy and Hbtot concentrations (Cui et al., 2011), decrease with improvements in response time, suggesting more efficient activation of PFC (Holland et al., 2011). Decreases in hemoglobin concentrations exist in the motor system (Hbdeoxy—Wolf et al., 2007), and in prefrontal cortex where they were correlated with reward value (Hboxy and Hbtot—DiStasio and Francis, 2013).

Although reported effects of primary motor cortex (M1) stimulation on skill acquisition and procedural learning have been promising, these methods have primarily been investigated in standard psychological and motor tasks including the serial reaction time task (Nitsche et al., 2003), the tower of London task (Dockery et al., 2009); and sequential visual isometric pinch task (Reis et al., 2009). Increasing evidence for the application of tDCS to enhance real-world skills has been reported for vehicle control (Beeli et al., 2008; Sakai et al., 2014), golf (Zhu et al., 2015), threat detection in image analysis (Falcone et al., 2012), air traffic control (Nelson et al., 2014). tDCS has also decreased resumption lag after interruption (Blumberg et al., 2014), and maintained vigilance (McIntire et al., 2014) in real-world tasks.

Critical for the acquisition of these real-work skills are both online and offline learning. Online learning is the change in behavioral performance across trials within an experimental session and is analogous to encoding (Reis et al., 2009). Offline learning is the change in performance, between sequential experimental sessions, from the last trial of the n-1th session to the first trial of the n^th^ session, and is analogous to consolidation (Robertson et al., 2004). The modulation of online and offline learning rate for practical, real-world skill acquisition with tDCS of M1 or DLFPC stimulation have remained unexplored.

Here, we investigated changes in skill acquisition and learning rates with tDCS applied to either DLPFC or M1 during custom pilot training exercises developed and administered with a commercially available flight simulator (X-Plane). These results were recently reported in a poster presentation at the Society for Neuroscience Meeting (Choe et al., 2015). We measured task-evoked changes in functional activity using fNIRS and EEG as subjects learned to complete flight simulator and N-back training exercises at increasing levels of expertise across four daily consecutive sessions. We hypothesized that stimulation of DLPFC over the course of flight simulation and N-back training would alter group variability in skill learning, MFT power, and Hboxy and Hbtot concentrations in the DLPFC. Furthermore, we hypothesized that tDCS of M1 will alter tonic alpha-band power over parietal cortex.

## Materials and methods

### Participants

Thirty-two right-hand dominant, healthy adult HRL Laboratories employees (31 males) participated in this study. Their ages ranged from 21 to 64 (mean ± STD = 38 ± 13). Participants were randomly assigned to one of four groups: DLFPC stim (*n* = 7, age = 35 ± 11), DLPFC sham (*n* = 7, age = 42 ± 13), M1 stim (*n* = 10, age = 41 ± 16), or M1 sham (*n* = 8, age = 31 ± 5). HRL Laboratories employees are a vulnerable class of subjects for this study. In order to manage the risk of any undue influence, coercion, or confidentiality breach we only allowed individuals who are not directly supervised by the investigators of this study to volunteer, and only performed experiments during normal business hours (9 a.m.–5 p.m.) to mitigate any possibility for recourse or reward for participation in performance evaluation or job advancement. To maintain confidentiality, each subject was assigned a unique number, known only to the investigators of the study and subject identities were not shared. This design is in line with the recommendations of Meyers (1979) on student and employees as a vulnerable population of subjects and complies with DHHS: protected human subject 45 CFR 46; FDA: informed consent 21 CFR 50. Inclusion criteria were: (1) normal or corrected-to-normal vision, (2) no prior history of epileptic seizures or known neurological disorders, and (3) no females who are pregnant or are likely to become pregnant during the course of the study. All participants provided written informed consent to participate in the experiment. JC, MDZ, and MEP are listed as inventors in patent applications on brain stimulation methods.

### Materials

#### Flight simulator

**Figure 1:**
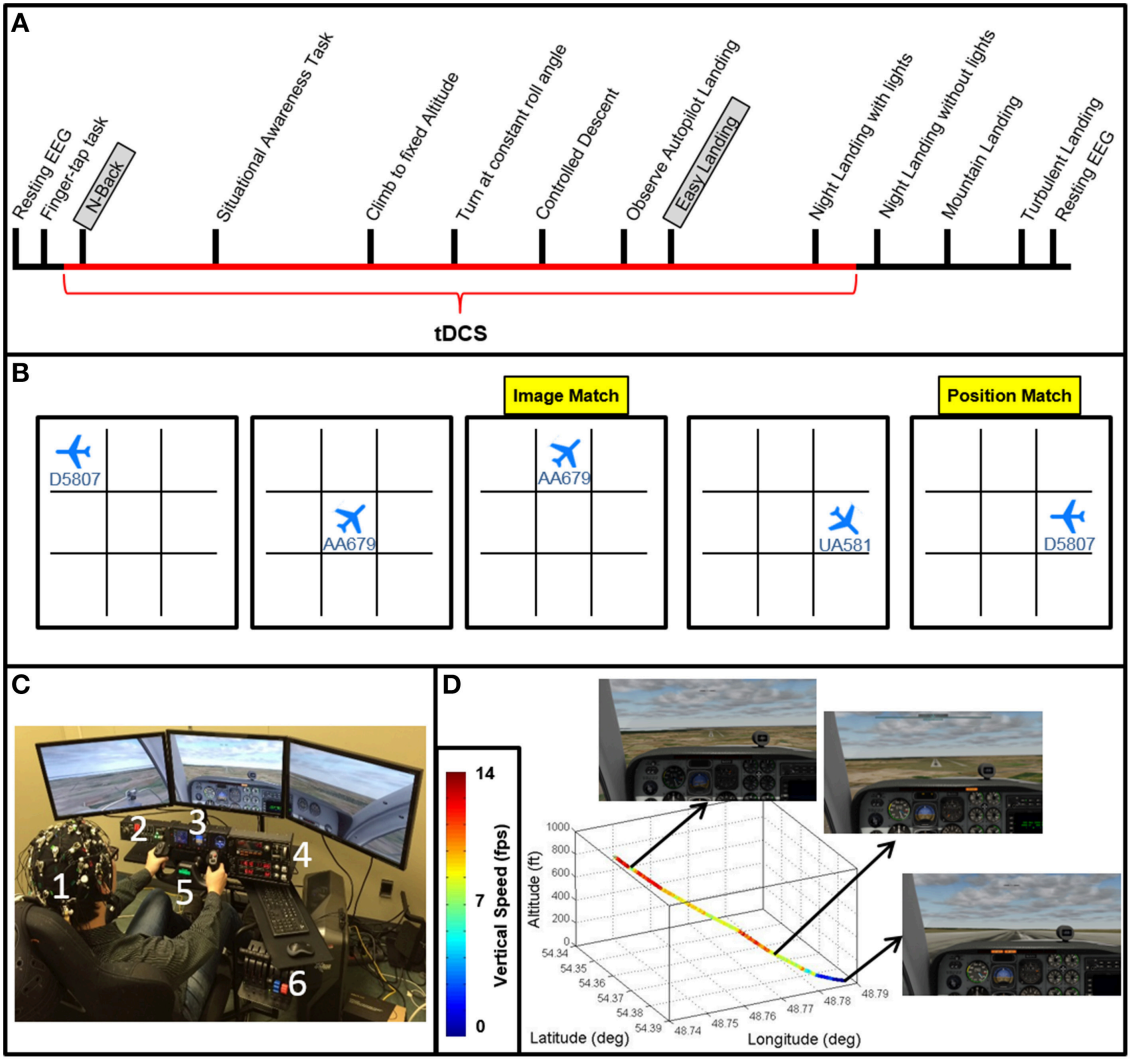
**Experimental design. (A)** Experiment timeline depicting the relative timing of each task (see Table 1 for descriptions of each task). The N-Back and Easy Landing tasks are highlighted, and the duration of tDCS is depicted in red. **(B)** An example of 6 trials of the N-Back task is shown. 1-back orientation and location match trials are highlighted in yellow. **(C)** The flight simulator, neuroimaging (EEG and FNIRS) and tDCS setup is shown with on a subject (1). Flight simulator equipment includes three-panel display, a radio panel (2), an instrument panel (3) with (from left to right) compass, altimeter, airspeed indicator, vertical speed indicator, and turn/slip indicator, a multi-panel (4) with (from left to right) autopilot settings, auto throttle switch, flaps switch, and elevator trim wheel, yoke (5), and throttle quadrant system (6). **(D)** Autopilot flight path for the Easy Landing task is shown in 3 dimensions, color-coded by vertical speed. Screenshots for initial descent, approach, and landing are also shown.

Flight simulation tasks were designed and administered with the XForce Dream Simulator package (X-Force PC) and the X-plane 10 Flight Simulator software (Laminar Research). A depiction of the XForce Dream Simulator package can be seen in Figure 1C, and included a yoke, a radio panel, an instrument panel with compass, attitude indicator, altimeter, airspeed indicator, vertical speed indicator, and turn/slip indicator, a multi-panel with autopilot settings, auto throttle switch, flaps switch, and elevator trim wheel, and a throttle quadrant system. This flight simulator included an adjustable seat for maximum comfort for the subject. Three monitors were placed at an optimal distance from the subject to avoid any eyestrain. Custom scenarios were designed using the simulator software development kit following a model of flight training (Williams, 2012, see Table 1).

**Table 1:** **Experimental task schedule performed on each of the four consecutive experimental sessions**.

**Task**	**# of trials**	**Task instruction/Description**	**Average duration (minutes)**
Survey	1	Setup and Explanation (day 1) Learn the basic controls via explanation	1
Rest	1	Eyes open, hand resting on yoke Observe straight and level autopilot flight	1
Finger tap	1	Using right hand, touch thumb to each finger in sequence	0.5
N-back	6	Adaptive threshold (at 80% accuracy) N-back on 3 × 3 grid of visual orientation of aircraft, flight number and spatial location (3 × 3 grid)	10
Situational awareness	4	Memorize gauge cluster images with 15 s distractor task and 30 s. for recall	10
Climb to fixed altitude	1	Adjust altitude from 5000 to 6000 ft. and back to 5000 ft. Maintain vertical speed at < 1000 ft./min with level roll	5
Turn at constant roll angle	1	Change bearing/azimuth from 90° to 180° and back to 90° with a maximum roll angle of 20°	5
Descend @ constant Rate	1	Adjust altitude from 3000 to 1000 ft. at a 800 ft./min rate of descent	5
Autopilot landing (observe)	1	Observe the autopilot approach on the “Easy Landing” runway in perfect weather and high visibility	2
Easy landing	5	Control all aspects of landing in perfect weather and high visibility	10
Nighttime landing	2	Control all aspects of landing in perfect weather at night, with runway lights (low visibility)	4
No-lights landing	2	Control all aspects in approach with zero visibility	4
Hard landing (Mountains)	1	Difficult visual-only approach over terrain where the runway is initially obstructed from view over a mountain range	6
Hard landing (Turbulence)	1	Control all aspects of landing with turbulence value is set to level 1 out of 10 (instead of 0), with high visibility	2

#### Neuroimaging

**Figure 2:**
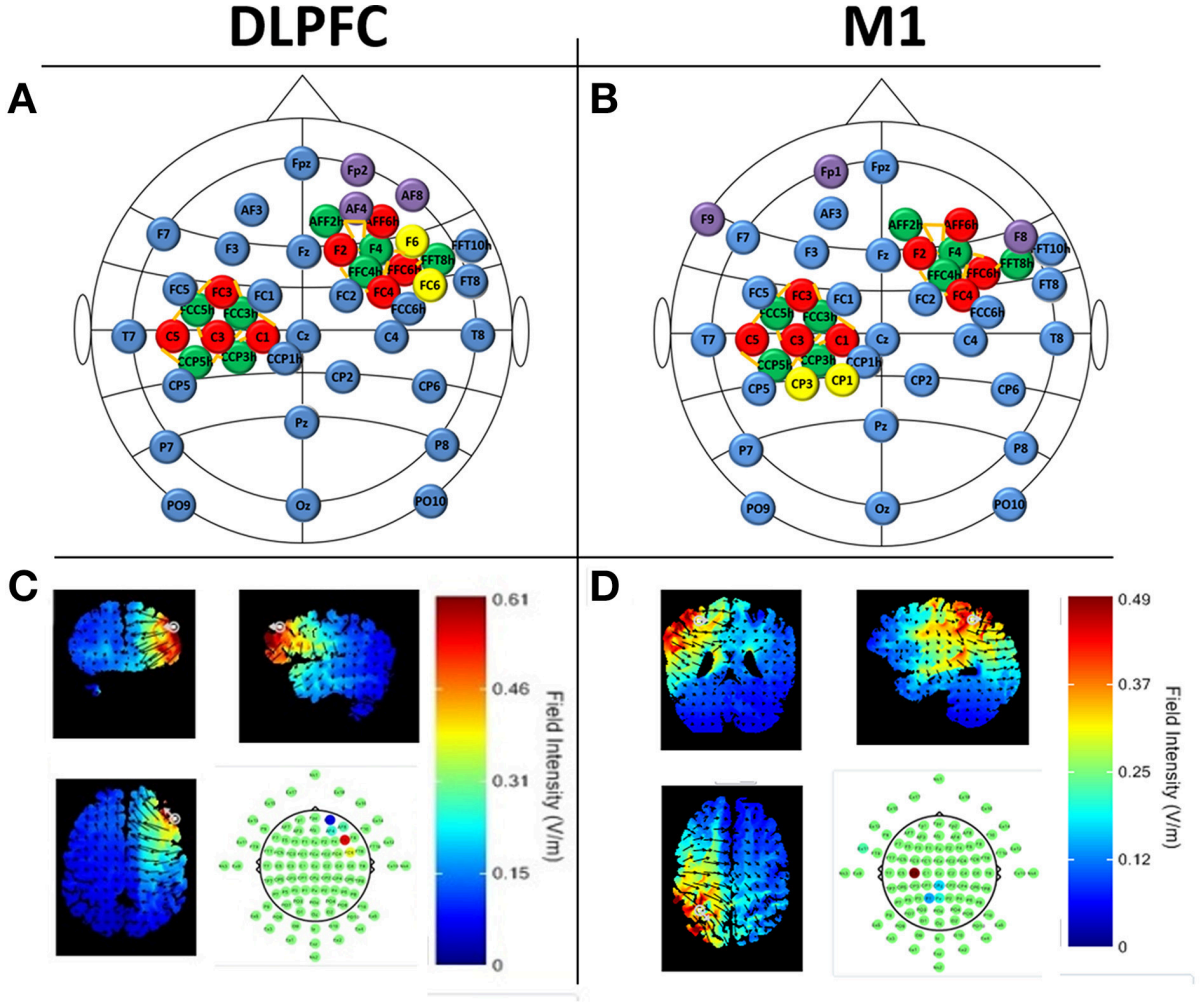
**Neuroimaging and tDCS experimental setup for DLPFC (A,C) and M1 stimulation (B,D). (A,B)** EEG locations are denoted in blue and follow the 10–20 locations where possible. fNIRS sources (red) and detectors (green) are shown over the left-M1 and right DLPFC with channels depicted as orange lines (M1 channels: FC3-FCC5h, FC3-FCC3h, C5-FCC5h, C3-FCC5h, C3-FCC3h, C1-FCC3h, C5-CCP5h, C3-CCP5h, C3-CCP3h, C1-CCP3h; DLPFC channels: AFF6h-AFF2h, AFF6h-F4, F2-AFF2h, F2-F2, F2-FFC4h, FFC6h-F4, FFC6h-FFT8h, FFC6h-FFC4h, FC4-FFC4h, FC4-F4) tDCS electrodes are denoted in purple (cathodes) and yellow (anodes) and follow the current values specified in Section Neuroimaging CandDBal prefrontal cortex (DLPFC [e confidence bound was >4x the size of the positive confidence bound]). Predicted electric field intensities from the maximum focality montages from the Male 1 model in the Soterix HD Targets software (Soterix Medical).

We recorded continuous EEG and fNIRS data during flight simulation training, N-back, finger tapping, situational awareness, and resting-state assessments. Horizontal and vertical electro-oculogram (EOG) was also recorded. EEG was collected using a 32-channel acti32Champ system, with electrodes placed in a custom, 10-10 based arrangement to accommodate tDCS electrodes (StarStim Neuroelectrics) and fNIRS illuminators/receivers (NIRSport NIRX) within custom headcaps (BrainVision). EEG caps were selected for each subject based on individual head size and aligned to Cz. Conductive gel (Signagel) was applied onto each EEG electrode and ultrasound gel (Aquasonic clear) was applied to each fNIRS source and detector. fNIRS was recorded with dual-wavelength continuous-wave (CW) near infrared (NIR) diffuse tomographic measurements at 760 and 850 nm. A total of 20 fNIRS channels (source-detector pairs) were recorded over the left M1 (10 channels) and right DLPFC (10 channels, see Figure 2). The distance between source-detector pairs was <3.5 cm (see Figure 2). EEG data were collected at 500 Hz, and fNIRS data were collected at 8 Hz. Locations of EEG electrodes and fNIRS channels can be seen in Figure 2.

#### tDCS

Sham or actual tDCS was applied with the Starstim system (Neuroelectrics) following the finger tapping task (see Figure 1A). The total current applied was 2 mA, with scalp current density of 0.04 A/m^2^ for active tDCS (for 60 min), or 0.1 mA (0.002 A/m^2^) for sham tDCS (for 1 min). Currents were applied with a 1 min ramp-in at initiation and a 1 min ramp-out at termination. Sham stimulation was used as a control condition to induce the physical sensation associated with tDCS (e.g., tingling) without directly stimulating the brain areas located below the electrodes (Coffman et al., 2012b). Silver/silver chloride electrodes were each 3.14 cm^2^ in size (total anode area = 6.28 cm^2^; total cathode area = 9.42 cm^2^). During stimulation the impedance value was limited to 20 kΩ for operation of the device; actual impedance values typically were below 10 kΩ and impedances were observed to be stable throughout the duration of the experiment. tDCS channel impedances were continually monitored at 1 HZ. To achieve maximum focality for the targeted brain regions of interest, electrode placements were derived using HD Targets (Soterix Medical) with stimulation targets in the left M1 (right posterior field orientation model) and right DLPFC (left anterior field orientation model) and possible electrode locations were defined using standard 10-10 electrode locations (see Figure 2). HD Targets uses a MRI-derived finite element brain model that provides predictions for current flow and alignment for multiple interacting electrodes; this model was used to calculate maximal focality and intensity for regions of interest. For M1 stimulation, this resulted in current values of: CP1 = 1244 μA, CP3 = 745 μA, FP1 = −417 μA, F8 = −448 μA, and F9 = −1124 μA. For DLPFC stimulation, current values were F6 = 1511 μA, FC6 = 482 μA, AF8 = −271 μA, AF4 = −283 μA, and FP2 = −1439 μA (see Figure 2). The predicted field intensities at the target locations were 0.56 V/m (DLPFC) and 0.45 V/m (M1). Groups are denoted as: DLPFC stim, DLFPC sham, M1 stim, and M1 sham.

### Procedures

All participants performed flight simulation training, N-back, finger tapping, situational awareness, and resting-state assessments once per day for four consecutive daily sessions (see Figure 1). Resting-state brain activity was collected for 1 min both before and after the experiment. During resting scans, subjects observed autopilot flight (level flight at 5000 ft. altitude) and were instructed to keep their eyes open and observe the visual scene while keeping their hands in their laps. Following the pre-experiment resting-state assessment, motor reference scans were taken during a simple motor sequence task in which subjects were instructed to touch each fingertip with the thumb of the right hand in sequence/cycle, continuously for 30 s (Figure 1A, finger-tapping task). We analyzed neuroimaging data recorded during the finger-tapping task as a confirmatory measure (see Supplementary Figures S4, S5), where sensorimotor network activity was expected to be evident in EEG as increased power in the beta band, and reduced power in the alpha band, compared to baseline, and in fNIRS as an increase in deoxygenated hemoglobin beneath M1 sensors.

Participants then performed the N-back task followed by a series of basic flight training exercises including a situational awareness task, climbing to fix altitude, turning at a constant roll angle, and a controlled descent. Follow these fight control tasks participants performed a series of landing task including the “easy landing” task, nighttime landing, nighttime landing without runway lights, a landing in mountainous terrain, and a landing in turbulent weather (Figure 1A). Results for the situational awareness, free flight, climb to fixed altitude, heading change at constant roll angle, descent at constant vertical speed, nighttime landing, no-lights landing, mountain, and turbulence landing task are the subject of subsequent manuscripts.

#### N-back

The Brain Workshop N-back task was implemented in this study (Paul Hoskinson, V.4.8.8 http://brainworkshop.sourceforge.net/). Participants monitored position and image for N-back matches without audio feedback. Custom N-back images were used, showing airplanes in eight different orientations with 1 of 3 possible flight numbers (24 total image possibilities, see Figure 1B). Subjects completed six blocks of 20 trials each day. Every subject began the N-Back task at the 1-back level, and was instructed at the beginning of each block to focus on a central fixation point. Subjects were free to move their eyes during the task. Upon reaching an upper threshold of accuracy within a given block (>80%), the task difficulty was increased (N + 1) using an adaptive threshold paradigm (Jaeggi et al., 2008). Upon reaching a lower threshold of accuracy (<20%), the task difficulty was decreased (N − 1). Each time this occurred, the changes were explained to the subjects between blocks. At the completion of each block, subjects were allowed to review the rules and ask clarifying questions about the tasks.

#### Autopilot landing observation

Subjects viewed a replay video of the autopilot executing an “optimal” landing from ~800 ft. altitude onto a runway. Initial aircraft position was aligned with the runway and aircraft was already maintaining proper vertical speed for ideal glide slope. This scenario presents a wide, long, flat runway with no visual obstructions and no landscape features that interfere with landing the aircraft. Subjects were instructed not to manipulate controls or control the simulation in any way, but were told to pay close attention to the flight parameters through the instrumentation, as well as the visual field displayed by the simulator as the aircraft proceeded with landing. Particular emphasis was placed on two key parameters: azimuth (20°) for runway alignment, and vertical speed (~700–800 ft./min) for appropriate glide slope. Attention was also drawn to the final control input to landing (pitch up at ground contact), and subjects were instructed to minimize landing force (G-force) as a top priority. Once the autopilot landing was viewed in its entirety, subjects were given the opportunity to ask questions about the landings. Most subjects asked very few, if any questions, typically on the first trial day.

#### Easy landing task

Subjects were instructed to complete the landing task as shown by the autopilot under daylight conditions and 100% visibility. Subjects attempted landing under these conditions a total of 5 times per day. As the subject attempted replication of the autopilot landing, the experimenter made observations in three categories: (1) Vertical speed maintenance; (2) Runway alignment; and (3) Final approach dynamics (pitch angle at touchdown). Any large deviations from the autopilot in any of these modalities were noted, then provided as feedback to the subject after the plane had touched down and the simulator paused. When given, feedback was ~1–2 min in length and conducted in an informal manner. The time duration of feedback also shortened throughout training as the subject made fewer errors. Following feedback, the subject was offered opportunity to ask any questions regarding landing technique, then the scenario was restarted. If subjects passed beyond the terminal end of the runway, the attempt was ended and the landing listed as “missed landing.” This counted against the number of subject attempts (i.e., attempts were not repeated due to missed landing). Feedback methods and handling of missed landings was identical for all landing task.

### Data analysis

#### EEG

EEG data were preprocessed using EEGLAB (Delorme and Makeig, 2004) by applying a 0.5 Hz high-pass filter (Butterworth, 12 dB/oct) and removing bad channels (max = 19%). Adaptive Mixture Independent Components Analysis (AMICA) (Delorme et al., 2012) was then used to detect and remove artifacts associated with eye blinks, vertical and horizontal electrooculogram, electrocardiogram, and tDCS-related voltage fluctuation. Following artifact rejection using AMICA, data were back-reconstructed and channels removed prior to AMICA decomposition were interpolated back into the data by spherical interpolation. Blocks corresponding to N-back, resting-state, and Easy Landing tasks were then segmented from the data.

Frequency decomposition was performed using FieldTrip (Oostenveld et al., 2011) by first segmenting data for each task into sequential 1-s epochs. Data were then windowed using a hanning taper, and frequency content of each trial was assessed at 1 Hz increments from 4 to 7 Hz (theta-band) or 8–12 Hz (alpha-band) using Fast Fourier Transform (multitaper method). After frequency decomposition, epochs with average theta or alpha power greater than two standard deviations from the mean were rejected, and remaining epochs were averaged for each participant, training day, and task. Data missing due to equipment issues (i.e., amplifier battery failure: *N* = 4, stimulus trigger errors: *N* = 1, or excessive noise/artifact during recording which could not be removed with AMICA: *N* = 7) were replaced with the mean for that participant group and training day prior to statistical analysis. We verified sensorimotor network activity during the finger-tapping task on the first day of flight simulator training (prior to tDCS) within baseline-subtracted beta and alpha band power maps, calculated across all subjects (Supplementary Figures S4, S5).

Participants receiving tDCS were compared with sham tDCS participants at each of the 4 days of training using independent-samples *t*-tests, which separately tested differences in alpha-band and theta-band activity at each sensor. Additionally, day 1 was compared to day 4 within each tDCS group and sensor using paired *t*-tests to assess training-related effects on alpha-band and theta-band activity. Statistical tests were corrected for multiple comparisons using cluster-based permutation tests (500 repetitions, data point α = 0.05, cluster-level α = 0.05, minimum spatial extent = 2 channels). Results from these comparisons are reported separately for each cluster of significant differences between groups/conditions. We calculated mean alpha/theta band power within clusters for use in examining relationships between task-related EEG and fNIRS/behavioral data.

We also examined correlations between behavioral measures, fNIRS beta values, and mean theta/alpha power across clusters identified during cluster-based permutation tests comparing days 1 to 4. fNIRS beta values were unavailable for 7 subjects (4 active M1 subjects and 3 sham M1 subjects) because time stamps could not be parsed from the fNIRS data files; therefore, the number of participants used in this analysis were: M1 stim = 6, M1 sham = 5, DLPFC sham = 7, and DLPFC stim = 7. These correlations were examined only within the stimulation groups where significant clusters were identified. To investigate relationships between midline frontal theta-band activity (Midline frontal theta-band activity was calculated as the mean theta power across electrodes Fz and FC1, the electrodes nearest to medial prefrontal cortex) and behavioral measures in the easy landing and N-Back task, Pearson correlation statistics were examined. We compared midline frontal theta-band activity in the easy landing task with autopilot displacement, g-force at landing, vertical speed at landing, roll at landing, pitch at landing, or online/offline learning rates for number of control inputs, autopilot displacement, vertical speed deviance from autopilot, or vertical speed variance. In the N-Back task, we compared midline frontal theta-band activity with average N level achieved and online/offline learning rates. Correlations were examined separately for DLPFC and M1 groups, stim and sham groups, and days of training. Because of the large number of correlation statistics examined, we used a conservative alpha of 0.001 to determine statistical significance. We additionally report statistics with a relaxed alpha of 0.05; however, these effects will be considered trends in this analysis.

In addition to cluster-based permutation tests across all channels, 3-way split-plot ANOVA was used to compare midline frontal theta-band activity between tDCS conditions (stim and sham), days of training (day 1, 2, 3, and 4), and training block (Block 1, 2, 3, 4, and 5) for the N-back and easy landing tasks. Huynh-Feldt epsilon was used to correct degrees of freedom for assumptions of sphericity, and Fishers Least Significant Difference corrections of alpha were used for simple-effects/pairwise comparisons (Maxwell and Delaney, 2004).

#### fNIRS

fNIRS data was processed within the nirsLAB analysis package (NIRx Medical Technologies, Glen Head, NY; Xu et al., 2014). The Gratzer Spectrum was used to measure the absorbance spectra of Hbdeoxy and Hboxy, with average wavelengths of 760 and 850 nm, respectively. The corresponding molar extinction coefficients ε are ε_Hboxy_ [1097.0 781.0] cm-1/M and ε_Hbdeoxy_ = [645.5 1669.0] cm-1/M, (nirsLAB, NIRx Medical Technologies). The differential path lengths were 5.98 for Hboxy and 7.15 for Hbdeoxy (Essenpreis et al., 1993). In the Beer-Lambert law calculation, the distance between source-detector pair was =< 3.5 cm, and the exact distances were computed within NIRSLab according to the corresponding distances on the headcap.

Hbdeoxy, Hboxy and Hbtot concentration time series were band-pass filter from 0.01 to 0.2 Hz (finite impulse response with least-squares error minimization), to remove slow drifts in the signal and respiratory and cardiac rhythms. Inter-trail data was removed from the time series, and the average baseline concentration values were subtracted from the task-evoked concentration measurements.

The average concentration value of Hbtot, Hboxy, and Hbdeoxy were computed separately for each channel, subject, task, and day. Concentration values were averages within days, across all 20 trails of each of the 6 blocks in the N-back, and all 5 trials of the easy landing task. Individual channel concentration values were then averaged across channels within regions (M1 and DLPFC) and across subjects within each group. Day1 group-averaged concentration values were then subtracted from Day 4 concentrations to compute the change in concentrations across the duration of the experiment.

Statistical significance of group-averaged concentrations changes from days 1 to 4 was determined using Statistical Parametric Mapping (SPM version 8). SPM was performed based on a general linear model of the canonical hemodynamic response function, with a discrete cosine transformation used for temporal filtering. A t-statistic-thresholded, baseline-subtracted Beta image was generated for each subject for baseline-subtracted, task-evoked Hbtot, Hboxy, and Hbdeoxy concentrations for days 1 and 4 (corrected for multiple comparisons across channels using the Bonferroni correction: # channels = 20, *p* < 0.0025). Paired t-statistic maps (subtracting the day 1 from day 4 betas) were generated from baseline-subtracted, trial/block-averaged (within day *n* = 5 Easy landing, *n* = 6 N-back) task betas obtained from individual subjects. If a t-statistic exceeded the corrected *p*-value threshold of 0.0025 the days 4–1 concentration values were determined to be significant (Table 2—denoted by bolded values).

**Table 2:** **Average ± standard deviation of day 1, day 4, and day 4–day 1 Hboxy, Hbdeoxy, and Hbtot concentrations across subjects and channels for M1 and the DLPFC**.

			**Easy landing**			**N-Back**		
			**Day 1**	**Day 4**	**Day 4–Day 1**	**Day 1**	**Day 4**	**Day 4–Day 1**
DLPFC Stim	M1	Oxy	−0.00060±0.0018	−0.0019±0.0013	**−0.0013 (8)**	0.00059±0.0010	−0.00041±0.00076	−0.00099
		Deoxy	−0.00037±0.00051	0.00012±0.0011	**0.0005 (4)**	−0.00017±0.00012	0.00021±0.00074	0.00039
		Total	−0.00098±0.0017	−0.0017±0.0012	**−0.00077 (8)**	0.00041±0.0010	−0.00019±0.00043	−0.00061
	DLPFC	Oxy	0.0030±0.0022	0.00055±0.0014	**−0.0024 (8)**	0.0021±0.0014	0.00048±0.00091	−0.0016
		Deoxy	−0.00024±0.00058	−0.00034±0.00041	−0.000095	−0.00018±0.00019	−0.00014±0.00021	0.000041
		Total	0.0027±0.0013	0.00021±0.0014	−0.0025	0.0019±0.0014	0.00034±0.00088	0.0016
DLPFC Sham	M1	Oxy	−0.0018±0.0020	−0.0018±0.0019	**−0.0000082 (10)**	−0.000094±0.0012	−0.000033±0.00098	**0.000062 (10)**
		Deoxy	−0.00022±0.00063	0.000065±0.0011	**0.00028 (4)**	−0.000099±0.00053	−0.00011±0.00022	**−0.00001 (4)**
		Total	−0.0020±0.0019	−0.0017±0.0012	**0.00028 (10)**	−0.00019±0.00077	−0.00014±0.00086	**0.000052 (10)**
	DLPFC	Oxy	−0.000033±0.0018	−0.00046±0.0014	**−0.00043 (1)**	0.00067±0.0012	0.00035±0.0012	**−0.00032 (4)**
		Deoxy	−0.00056±0.00043	−0.00053±0.00021	0.000035	−0.00034±0.00020	−0.00036±0.00013	**−0.000017 (8)**
		Total	−0.00059±0.0019	−0.00099±0.0013	**−0.0004 (4)**	0.00032±0.0012	−0.000015±0.0012	**−0.00034 (4)**
M1 Stim	M1	Oxy	0.00024±0.0031	−0.000084±0.0015	**−0.00032 (4)**	0.0013±0.0017	0.00045±0.00088	−0.00084
		Deoxy	−0.00019±0.0014	−0.00049±0.00028	**−0.0003 (5)**	−0.00048±0.00045	−0.00027±0.00031	0.00021
		Total	0.000043±0.0039	−0.00057±0.0014	−0.00062	0.00080±0.0016	0.00017±0.00085	−0.00063
	DLPFC	Oxy	0.00050±0.0026	−0.00060±0.00055	−0.0011	0.00015±0.00051	−0.00031±0.00036	**−0.00046**
		Deoxy	−0.000045±0.00092	−0.00017±0.00020	−0.00012	−0.00027±0.00030	−0.00012±0.000088	0.00015
		Total	0.00046±0.0035	−0.000766±0.00056	−0.0012	−0.00012±0.00078	−0.00043±0.00035	**−0.00031**
M1 Sham	M1	Oxy	−0.00097±0.0010	−0.00080±0.00085	**0.00017 (10)**	0.00022±0.0012	0.00047±0.0016	0.00025
		Deoxy	−0.00017±0.00020	−0.00013±0.00021	**0.000032 (8)**	−0.00015±0.00016	−0.00018±0.00020	−0.000031
		Total	−0.0011±0.00091	−0.00093±0.00089	**0.0002**	0.000074±0.0012	0.00029±0.0017	0.00022
	DLPFC	Oxy	−0.00041±0.00087	−0.00054±0.0019	−0.00013	0.00011±0.00030	−0.00030±0.00034	−0.00041
		Deoxy	−0.000019±0.00038	0.00084±0.0013	0.00086	−0.000087±0.00019	0.00026±0.00071	0.00035
		Total	−0.00043±0.0011	0.00030±0.0032	0.00073	0.000022±0.00029	−0.000043±0.00041	−0.000065

*All values are presented are in mM concentration units. Bold numbers indicate a significant difference between days 4 and 1 as determined by a SPM (see Materials and Methods Section fNIRS)*.

Channel-wise statistical analysis was performed on all channels for measurements of Hboxy, Hbdeoxy, and Hbtot days 4–1 concentrations in easy landing and N-back for all subjects. Significance was determined if the trial-wise average exceeded 3.5 standard deviations from the null hypothesis of no concentration change (Bonferroni corrected, two-sided, Fischer's test *p* < 0.00035).

#### Behavioral performance

##### N-back

Raw percent accuracy values for each subject and for each block were scaled according to the information content required for each back condition. A 100% score on a 1-back trial requires both an image match: 9 possible plane orientations, 4 possible flight numbers, and a position match: 9 possible spatial location, a 100% score on 2-back doubles the required information kept in working memory, and a 100% score on a 3-back trial triples this value. The normalization weights used for the 1–, 2–, and 3–back raw accuracy values were therefore 0.33, 0.66, and 1.0. Alternative normalization schemes (e.g., bit-wise maximum information and log-scaling) did not generate substantial differences in the outcome metrics. Learning rates were determined by the slope (±standard deviation) of a linear regression over block-wise group-averaged scaled percent accuracy: (1) across all 4 days (overall learning rate), (2) within each day independently (online learning rate), and (3) between the accuracy of the first trial of the nth day and the last trial of the n-1th day (offline learning rate) (Reis et al., 2009). Meta-learning rate was determined from the slope of the linear regression over the combined online/offline learning rate time series (the rate of change in the learning rates over time). The average number of trials for each group to reach the 2 and 3 back levels in the adaptive N-back task and the average streak (number of consecutive trials) at 2 and 3 back were calculated for each group. Learning rates were compared using one-sample against zero or paired, two-tailed *t*-tests (both α = 0.05) were noted.

##### Easy landing

*G-force assessment*. Flight parameters were sampled from the simulator at 10 Hz, including altitude (above ground level), longitude, and latitude. The derivative of the vertical speed of the aircraft at runway touchdown determined the landing impact g-force (acceleration divided by 9.8 m/s^2^). Smaller g-force landings reflected improved skill with the landing task as subjects were asked to minimize this value to the best of their ability for each trial. The impact g-force is a “one-shot” assessment of landing skill at the most difficult and critical phase of the landing task, while ignoring other factors of landing performance (e.g., approach, glide slope, alignment, aircraft attitude). Online, offline, and meta learning rates are negative indicating a reduction in the applied G-force at landing (Supplementary Table S2, **Figure 4**).

*Flight path deviation*. Latitude, longitude, and altitude were transformed into Cartesian (X-Y-Z) coordinates and the Euclidean distance between coordinates of subject and autopilot were computed over the flight path Σsqrt(x^2^+y^2^+z^2^) using a moving window average to resample and align the flight paths. The Euclidean distances for each sample were then summed in order to provide the total deviation from the autopilot flight path. Unlike G-force, this metric takes into account the entire approach, including all flight maneuvers leading up to the final descent and touchdown. This measure, however, does not take into account proficiency with aircraft controls or avionics; it merely assesses the ability of the subject to adhere to the reference flight path. Subjects were instructed to replicate the flight path of the autopilot landing observation. With this metric, a better landing would have lower deviation values (Supplementary Table S2, **Figure 5**).

*Vertical speed deviation*. The vertical speed of the subject throughout the landing trial was subtracted from the vertical speed of the autopilot landing at each time step and summed as in the flight path deviation. The vertical speed profile of the aircraft is stereotypic for an excellent landing and this parameter is visible on the aircraft's instruments. Subjects could therefore be reasonably expected to match the vertical speed of their aircraft with that of the example shown during autopilot observation (vertical speed maintained at 600 ft./min for majority of approach, see Figure 1D). Replication of the autopilot-derived demonstration flight should result in lower overall vertical speed deviation values as performance improves (Supplementary Table S2, **Figure 5**).

*Vertical speed variance*. The amount of vertical speed variation throughout the landing approach was summed across trials to represent the degree to which a subject could maintain a steady, continuous descent. This measure does not penalize the subject for deviating from the ideal flight path, it merely assesses the degree to which the subject can maintain a smooth descent with little variation. This removes the goal-directed aspect of flight parameter maintenance while focusing on the motor aspect of flight parameter maintenance. As a means of comparison, the autopilot flight data only changes vertical speed in the final 5 s before landing, which minimizes this variance in the autopilot. In the ideal scenario, vertical speed stays constant, with only slight changes necessary for the final phase of landing; therefore, smaller variances indicate superior flight performance (Supplementary Table S2, **Figure 6**).

*Control input measure*. The number of control inputs was computed over landing trials by identifying the number of sign-changes in the vertical speed parameter throughout the landing period. This metric identifies to what extent subjects could maintain a consistent vertical speed profile (negative vertical speed indicates descent, positive ascent). Since maintaining vertical speed with minimal control input adjustment does not require specific planning of actions or prediction of flight path, it was hypothesized to be a primarily motor-processing focused measure. The number of sign-changes in the vertical speed variable was summed between start and end of the landing. As a means of comparison, the autopilot had 1 major control input at the nose flair ~1 s. before touchdown.

##### Outlier rejection

For each metric, trial-wise data were examined for outliers across subjects across all groups. If any trial exceeded three standard deviations from the mean, it was determined an outlier and removed from analysis. Outlier rejection was performed on a trial-wise basis for all computed metrics.

##### Group variance analysis

For each metric, the variance in the average online learning rate was computed as the change in the group's average accuracy treating each subject's performance in a trial as a repeated sample within days. For the n-back task, the metric used was the scaled percent accuracy across 6 trials per day. For the easy landing task, flight metrics, performed over the course of 5 landing trials per day. This measure is the variance in the online learning rate linear regression (Reis et al., 2009). Significant differences in learning rate variance was assessed with a two-sample *F*-test for equal variances. The null hypothesis that two independent samples of two subject pools come from a single normal distribution with the same variance was tested against the alternative that they come from two normal distributions with different variances. F-stat criticality was computed by generating a F cumulative distribution function appropriate to the variance ratio and degrees of freedom of sample pools. The resulting critical values are asymmetric and can be used at either tail. We were then able to determine the distance between computed F and *F* = 1 (null hypothesis). Across days, Bartlett's test was performed to test the hypothesis of equal population variance across groups. This test was performed on the average subject metrics (across trials within days) to preserve sample independence. Reported *p*-values represent the probability of observing the given result by chance if the null hypothesis were true (Snedecor and Cochran, 1989).

## Results

### Finger-tapping task

As expected, the finger-tapping task induced beta band oscillatory activity and increased the concentration of Hbdeoxy over sensorimotor cortex, and reduced alpha band activity over frontal and parietal cortex compared to baseline (Supplementary Figures S4, S5). Power in the beta band was greatest over left sensorimotor cortex, contralateral to the hand used during the finger-tapping task.

### N-back task

#### Behavioral results

##### DLPFC stimulation

**Figure 3:**
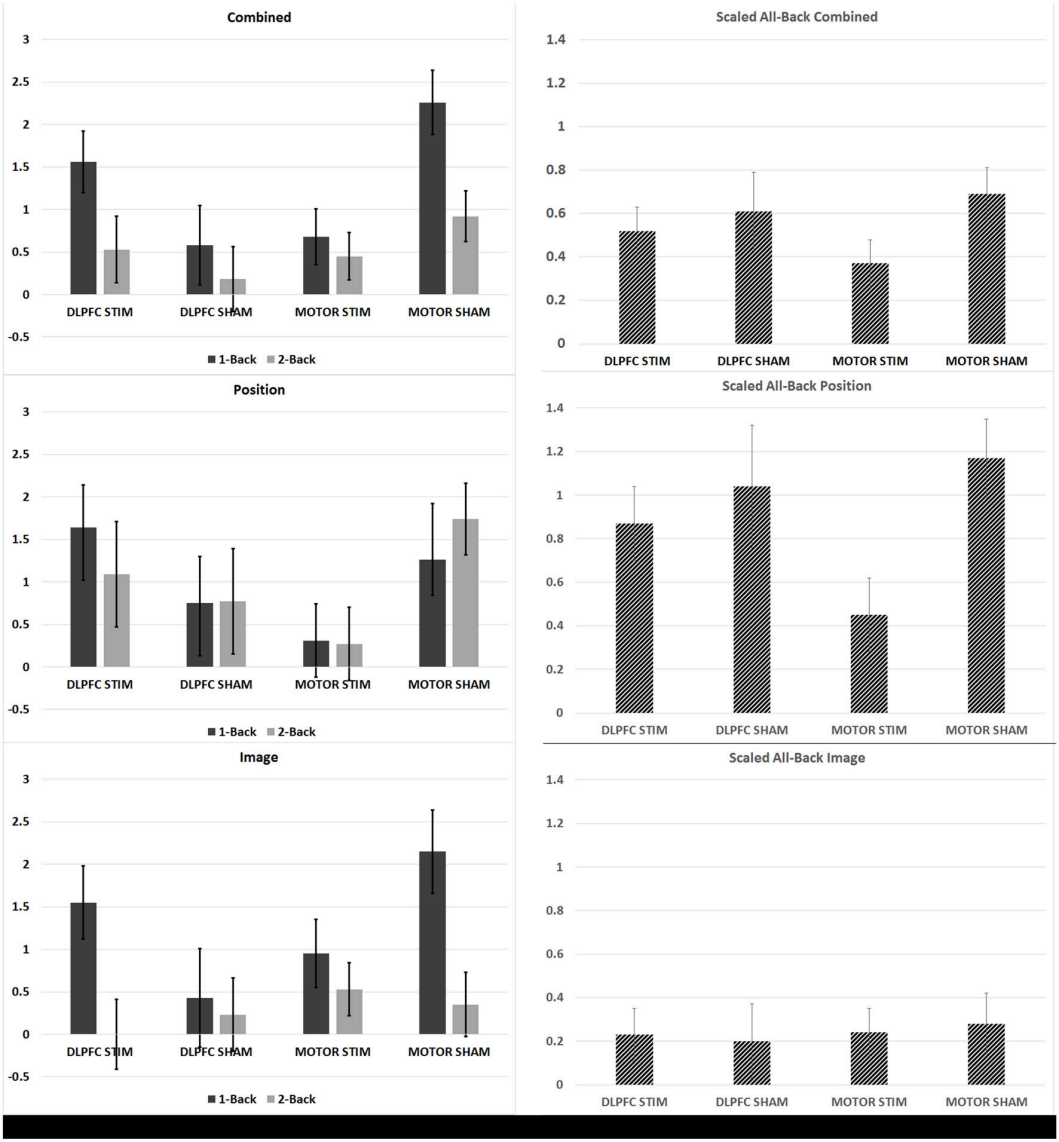
**N-back learning rates across experimental groups**. The average group-learning rate is shown for each group in 1 and 2 back trials (left) and for all back trials (right column scaled by information content see Section N-Back). Learning rates computed by combining across position and image match trials **(top row)**, for position trails **(middle row)** and image trials **(bottom row)** are shown.

**Figure 4:**
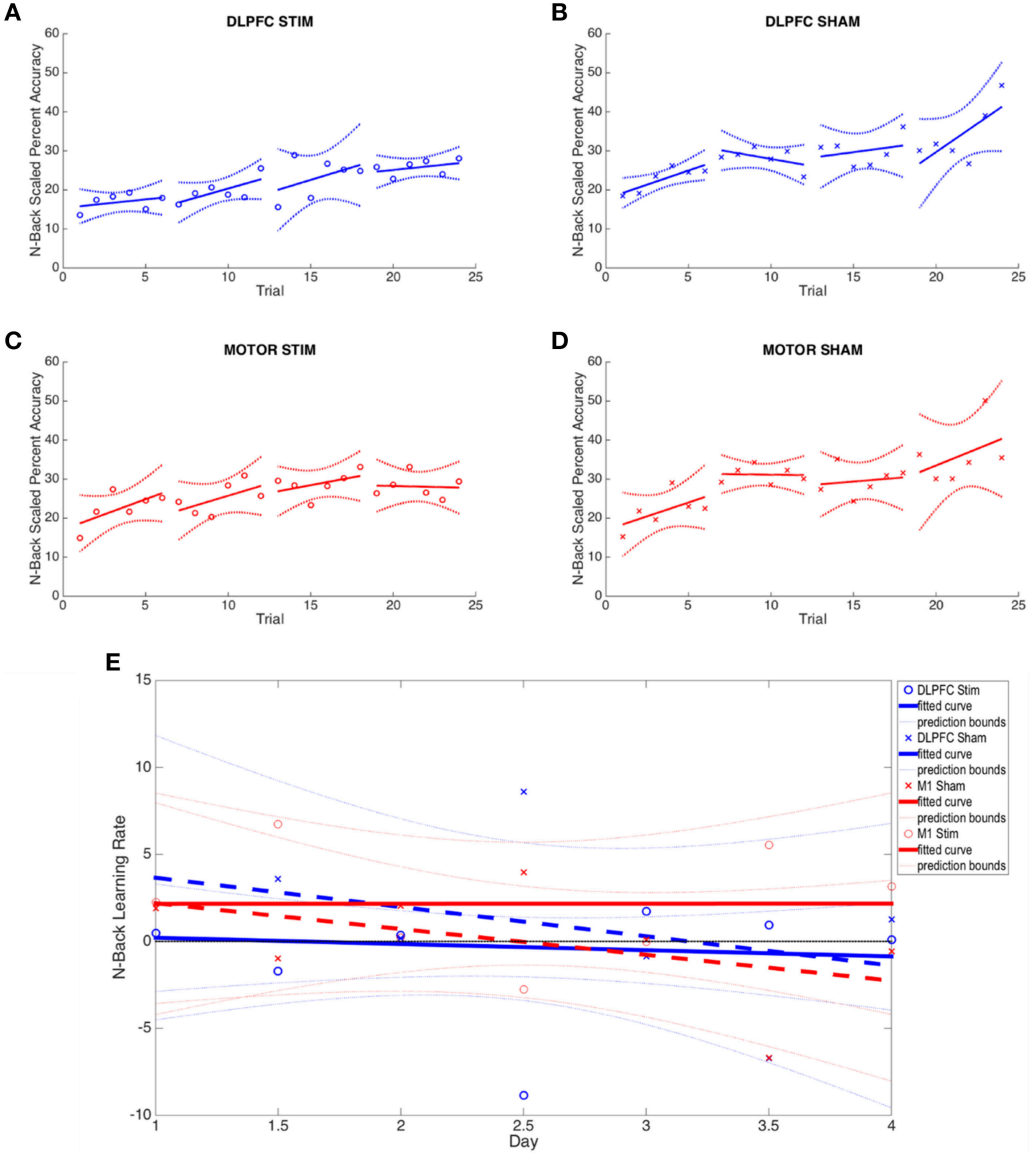
**N-back results across all four experimental groups. (A–D)** The average percent correct is plotted across all 4 days (6 trials per day) for each group. Percent correct values are scaled based on the required information for a 100% correct response (see Section N-Back). **(E)** Online and offline N-back learning rates are plotted for each experimental group across the duration of the experiment. Whole numbers on the x-axis represent the average online learning rate (slope of scaled percent correct linear regression for each subject across 6 blocks within a day and numbers of the x-axis represent offline learning rates (slope of the percent correct on the last trials of the N-1 day to the first trial of the Nth day).

The DLPFC stim group showed significant overall learning in five separate learning rate measures, compared to two significant learning rates observed for the DLPFC sham simulation group (one-sample, two-tailed *t*-test, see Figures 3, 4, and Supplementary Table S1). Significant overall learning was observed for the DLPFC stim group collectively across all trial types (combining image and position match trials—denoted as “combined trials,” and for position match trails) aggregating across all 1/2/3 back trials (scaled according to the methods in Section N-Back). Significant overall learning was also observed for the DLPFC stim group in 1-Back combined, position, and image trials. Significant overall learning was observed for the DLPFC sham group for combined and position trials, across all backs (see Figure 3). Meta-learning regressions did not show statistically significant changes in learning rates between stim and sham groups.

Neither the initial nor the final behavioral performance were significantly different between DLPFC stimulation and sham groups (Supplementary Table S1). The average trial duration to reach 2-/3-back was not significantly different between stimulation and sham groups. In addition, the average number of trials to reach 2-/3- back and the average 2-/3- back streak durations were not statistically different between groups. Significant differences in online, offline, and combined learning rates were not observed between stimulation and sham groups (see Supplementary Table S1, Figure 4E).

The variance in the DLPFC stim group's learning rate was significantly less than the variance of the DLPFC sham group. Examined across days, the DLPFC stim group had significantly reduced variance compared to the DLPFC sham group on Day 3 of experimentation [Chi_(1)_ = 5.77, *p* < 0.02, see Figures 4A,B]. Examined at the trial-level, the reduced variance reached statistical significance in >33% of individual N-back trials comparing DLPFC stim with DLPFC sham, and no trials showed greater variance in the DLPFC sham group [Day 1 Trial 2: *F*_(6, 6)_ = 0.21; Day 1 Trial 4: *F*_(6, 6)_ = 0.18; Day 1 Trial 6: *F*_(6, 6)_ = 0.23; Day 2 Trial 5: *F*_(6, 6)_ = 0.095; Day 3 Trial 1: *F*_(5, 5)_ = 0.069; Day 3 Trial 2: *F*_(5, 5)_ = 0.17; Day 3 Trial 6: *F*_(5, 5)_ = 0.15; Day 4 Trial 6: *F*_(5, 4)_ = 0.021; *p* < 0.05]. These results support the hypothesis that tDCS of the right DLPFC would reduce the variability in individual learning rates in a cognitive task.

##### M1 stimulation

The M1 stim group showed significant overall learning in five separate learning rate measures, compared to six significant overall learning rates observed for the M1 sham stimulation group. Significant overall learning was observed for the M1 stim group for combined, image and position trails aggregating across all 1/2/3 back trials. Significant overall learning was also observed for M1 stim in 1-Back combined and image trails. Significant overall learning was observed for the M1 sham group for combined trails, aggregating across all backs and for 1 and 2-Back trials as well as for position trails (all and 2-Backs) and 2-Back image trials (see Figure 3). Meta-learning regressions did not show statistically significant changes in learning rates between stim and sham groups.

As with the DLPFC groups, initial and final behavioral performance between stimulation and sham groups was not significantly different (Supplementary Table S1). The average duration to reach 2-/3-back was not significantly different between stimulation and sham groups. In addition, the average number of trials to reach 2-/3- back and the average 2-/3- back streak durations were not statistically different between groups. Significant differences in online, offline, and combined learning rates were not observed between stimulation and sham groups (see Supplementary Table S1 and Figures 3, 4E).

Unlike the results observed for DLPFC stimulation, M1 stimulation resulted in minimal differences in learning rate variance between stimulation and sham groups. Only 1 trial showed reduced M1 stim variance compared to M1 sham variation, while there were 3/24 trials that indicated smaller M1 sham variance when compared with the values from the M1 stim group. Examined across days, no trials shows significant differences in variance under Bartlett's Test.

#### FNIRS results

##### DLPFC stimulation

*Hboxy*. Exclusion criterion for individual FNIRS channels were greater than 0.001 mM fluctuations between the maximum and minimum measured concentrations during baseline. We did not observe any concentration fluctuations above this cutoff threshold for any of the 20 FNIRS channels (10 above the DLPFC and 10 above the M1 cortex) across all 4 days of recording for the 25 subjects analyses (7 DLPFC stim, 7 DLPFC sham, 6 M1 stim, 5 M1 sham). Subjects were not included (*n* = 3 in M1 stim, and *N* = 3 in M1 sham) if event time stamps could not be identified robustly within the fNIRS data files.

Average Hboxy concentrations across subjects and channels significantly increased between day 1 and day 4 in M1 channels, and significantly decreased in DLPFC channels for the DLPFC sham group and (see Table 2, **Figure 7**). Individual channel analysis showed no significant change in Hboxy concentrations from days 1 to 4.

*Hbdeoxy*. Average Hbdeoxy concentrations across subjects and channels significantly decreased between day 1 and day 4 in M1 and DLPFC channels for the DLPFC sham group (see Table 2).

*Hbtot*. Like Hboxy, average Hbtot concentrations across subjects and channels significantly increased between day 1 and day 4 in M1 channels for the DLPFC sham group, and significantly decreased in DLPFC channels for the DLPFC sham group (see Table 2). Individual channel analysis showed no significant change in Hbtot concentrations from days 1 to 4.

##### M1 stimulation

*Hboxy*. The average Hboxy concentration across subjects and channels within the DLPFC channels significantly decreased between days 1 and 4 in the M1 stim group (see Table 2). Individual channel analysis shows no significant change in Hboxy concentrations from day 1 to 4.

*Hbtot*. The average Hbtot concentration across subjects and channels within the DLPFC channels significantly decreased between day 1 and 4 in the M1 stim group (see Table 2). Individual channel analysis shows no significant change in Hbtot concentrations from days 1 to 4.

#### EEG results

##### Theta (4–7Hz)

*DLPFC stimulation*. In each day, significant differences in theta-band power were found between DLPFC stim and sham tDCS groups in frontal/central electrodes (Table 3). In days 1–3, right frontotemporal theta power was higher in DLPFC stim participants, compared to sham. Statistical differences were distributed over midline frontal electrodes in day 4. Comparison of days 1 and 4 revealed a significant increase in midline frontal theta-band power in stim, but not sham participants (see **Figure 8A** and Table 3). Split-plot ANOVA comparing MFT in the N-back task revealed a trend-level main effect of tDCS group, with DLPFC stim participants showing a greater effect than DLPFC sham participants [*F*_(1, 12)_ = 4.65, *p* = 0.052]. There was no main effect of training or interaction between tDCS group and day of training (*p* > 0.1).

**Table 3:** **Cluster statistics for comparisons of alpha- and theta-band power during the N-back task**.

	**Electrodes**	t^*^	p^**^
**THETA (4–7 Hz)**			
**DLPFC stimulation** *Actual vs. Sham* Day 1 Cluster 1	E5, FT8, T8, FC2, CP2, CP6	2.89	0.020
Day 2 Cluster 1	FT8, FC2, E11, CP2, CP6, PO10	3.15	0.006
Day 3 Cluster 1	FT8, E11, T8, CP6	3.11	0.020
Day 4 Cluster 1	AF3, F3, Fz	3.21	0.038
*Day 4 vs. Day 1* Actual Cluster 1	FC1, Cz, E19	3.23	0.020
**M1 stimulation** *Actual vs. Sham* Day 1 Cluster 1	FC5, FC1, T7, Cz, C4, T8, E19, CP2, P7, Pz, P8, PO10	2.82	0.004
Day 3 Cluster 1	E5, T8, Cz, CP5, CP2, CP6, P7, Pz	2.27	0.020
Cluster 2	E5, FC2, C4, CP6	2.35	0.028
**ALPHA (8–12 Hz)**			
**DLPFC stimulation** *Actual vs. Sham* Day 2 Cluster 1	E5, FT8, E11, T8	2.85	0.008
Cluster 2	AF3, F7, F3	2.69	0.042
Day 4 Cluster 1	Pz, P8, Oz	2.74	0.048
Day 4 vs. Day 1 Actual Cluster 1	FC5, T7, CP5, P7	−3.12	0.032
**M1 stimulation** *Actual vs. Sham* Day 1 Cluster 1	F3, Fz, FC5, FC1, FC2, Cz	2.88	0.014
Day 3 Cluster 1	FC5, T7, CP5, P7	2.66	0.034

* *Reported t-values are the average t-statistic across all electrodes in a given cluster*.

** *Reported p-values are corrected for multiple comparisons using cluster-based permutation tests*.

*M1 stimulation*. For M1 stimulation, broadly-distributed differences in theta-band power were seen between stim and sham participants during N-Back performance on days 1 and 3, which were mostly left-lateralized, and were strongest near the site of stimulation (Table 3). Importantly, no differences between days 1 and 4 were seen for M1 stim or sham participants in the N-Back (see Table 3 and **Figure 8A**). Split-plot ANOVA comparing MFT revealed a significant main effect of day of training [*F*_(3, 48)_ = 3.23, *p* = 0.048]; however no significant or trend-level pairwise comparisons were found, There was no main effect of tDCS group or interaction between group and day of training.

##### Alpha (8–12Hz)

*Dlpfc stimulation*. Alpha-band power differences between DLPFC stim and sham groups were found in days 2 and 4 (Table 3). In day 2, frontal alpha power was greater in DLPFC stim than sham participants. In day 4, differences existed in parietal and occipital electrode sites, with DLPFC stim greater than sham. Differences between day 1 and 4 were found only for the DLPFC stim group, characterized by reduced alpha power at left temporoparietal sites (see Table 3 and **Figure 8B**).

*M1 stimulation*. Greater alpha-band power was found for M1 stim compared to sham participants in days 1 and 3 (Table 3). These differences were distributed over frontal, central, and parietal electrode sites, mostly near the site of stimulation. No differences in alpha power were found in the comparison of days 1 and 4, for either M1 stim or M1 sham (see Table 3 and **Figure 8B**).

##### EEG/fNIRS/behavioral correlations

We did not find any significant correlations between MFT or alpha power in the N-Back task and behavioral measures (i.e., average N level achieved and online/offline learning rates, *p*'s > 0.05). No correlations were identified between fNIRS beta values and either MFT or alpha power in the N-Back task for any group (*p*'s > 0.1).

### Easy landing task

#### G-force

##### DLPFC stimulation

**Figure 5:**
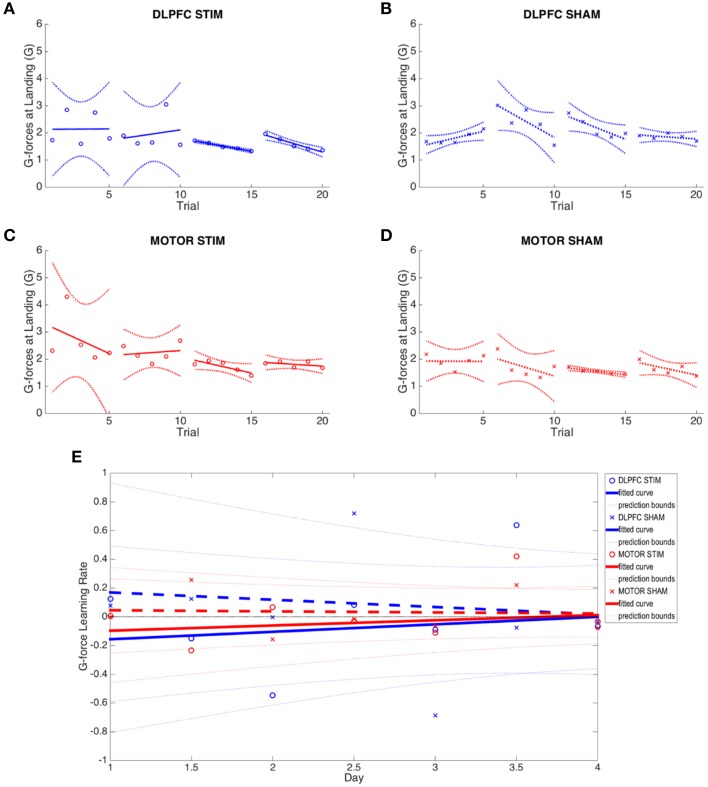
**G-force at moment of landing results across all four experimental groups. (A–D)** Average g-force at moment of landing across all 4 days is plotted for each group. Note reduction in between-subject variance in days 3 and 4 of the DLPFC stim group. **(E)** Online and offline g-force learning rates are plotted for each experimental group across the duration of the experiment. Whole numbers on the x-axis represent the average online learning rate (slope of scaled percent correct linear regression for each subject across 6 blocks within a day) and numbers on the x-axis represent offline learning rate (slope of the percent correct on the last trials of the N-1 day to the first trial of the Nth day). Smaller G-force indicates improved performance.

tDCS to DLPFC reduced the variability (standard deviation) of the third and fourth day online learning rates compared to sham (DLPFC stim: day 3 = 0.355, day 4 = 0.583; DLPFC sham: day 3 = 0.846, day 4 = 0.637, see Figure 5). First trial comparisons of variance between DLPFC stim and DLPFC sham groups in day 3 showed statistically significant changes in between-subject variance [*F*_(4, 5)_ = 0.046, *p* < 0.02]. There were no significant differences in variance for first trials of day 1 and day 2 (*p* > 0.1). Trial 1 of day 4 also did not show significant changes in variance (*p* > 0.1). Examined across days, Bartlett's comparisons of variance between DLPFC stim and DLPFC sham groups in day 3 showed statistically significant changes in between-subject variance [Chi_(1)_ = 7.33, *p* < 0.01]. There were no significant differences in variance for days 1, 2, or 4 (*p* > 0.1). These results support the hypothesis that tDCS of the right DLPFC would reduce the variability in individual learning rates in the easy landing task.

Learning rates were determined by computing the rates at which performance improved (i.e., reduction of G Force over time, see Figures 5A–D). Meta-learning regressions did not show statistically significant changes in learning rates between stim and sham groups. The DLPFC stim group exhibited positive meta-learning rates (DLPFC stim = 0.052 ± 0.090), where the DLPFC sham group, by contrast, showed overall negative meta-learning (DLPFC sham = −0.051±0.106), but this between-group difference did not reach statistical significance due to large within –group variance (*p* > 0.1; Figure 5E).

There were also no statistically significant differences in offline learning rates (*p*'s > 0.1), but DLPFC stim showed a relatively strong offline learning between day 1 and 2 (−0.149 ± 0.52) compared to sham (0.124 ± 0.990).

The number of missed landings (did not land before or during the runway) was not different across groups over days (DLPFC stim: day 1: 2.9%, day 2: 2.9%, day 3: 0%, day 4: 0%; DLPFC sham: day 1: 2.9%, day 2: 14.3%, day 3: 5.7%, day 4: 0%). Missed landings typically occurred on the first trial of the day.

##### M1 stimulation

tDCS to M1 resulted in no significant changes in inter-subject variance when compared to the M1 sham group across days (Bartlett's Test, *p*'s > 0.1). M1 sham appeared to have unusually low variance during day 2 (Figure 3D), and this was determined to be a statistically significant reduction of variance when compared to the M1 stim group for three of the five trials of Day 2 [*F*_(8, 7)_ = 6.32, *F*_(8, 7)_ = 3.86, *F*_(8, 7)_ = 5.07; *p* < 0.05]. However, this reduction in variance only applied to Day 2 and in single trials only in Day 1 and 3. All trials on Day 4 had no significant change between M1 stim and sham variances. There were no statistically significant changes in learning rates between stim and sham groups (Supplementary Table S2). As with DLPFC stim group, there were also no statistically significant differences in offline learning rates (*p*'s > 0.1), but M1 stim showed a relatively strong offline learning between day 1 and 2 (−0.235 ± 0.676) compared to sham (0.258 ± 1.330).

Initial starting (day 1 average), group averaged (across all days and the final (day 4 average) G-forces were not significantly different between experimental and control groups (paired *t*-test *p* > 0.1), which are similar to the results found with the N-back task. Though performance improved in both sham and stimulation cohorts (reduced overall G-force), the ultimate performance of each subject group was similar. It is probable that the landing task was effectively learned over the course of four training days, and subjects reached a G-force performance ceiling.

The number of missed landings were not different across groups over days (M1 stim: day 1: 10%, day 2: 10%, day 3: 2.0%, day 4: 0%; M1 sham: day 1: 0%, day 2: 0%, day 3: 0%, day 4: 2.5%).

#### 3D autopilot displacement

**Figure 6:**
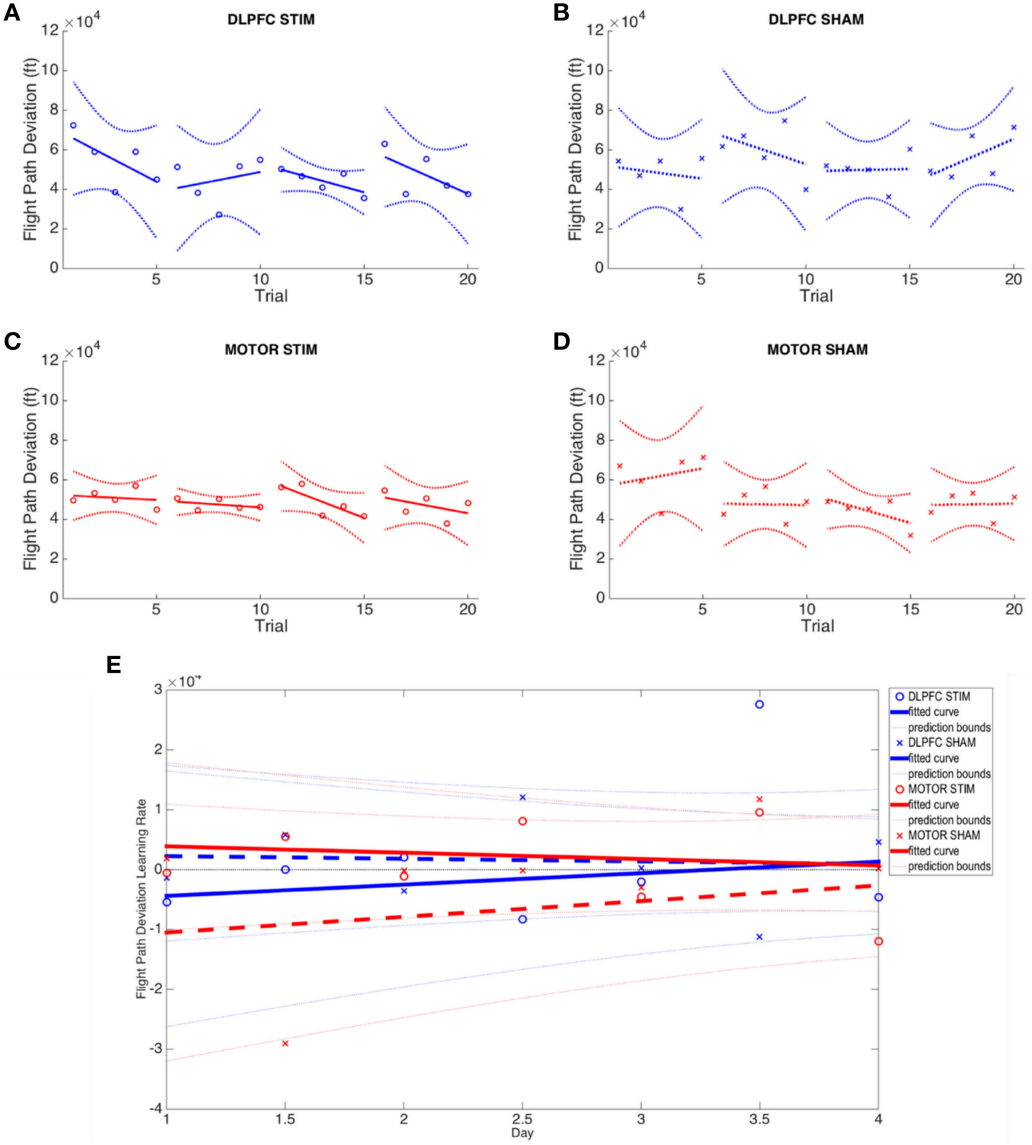
**Flight path deviation results across all four experimental groups. (A–D)** Average flight path deviation of subjects from ideal autopilot-guided glide slope is shown for each group. **(E)** Online and offline flight path deviation learning rates are plotted for each experimental group across the duration of the experiment. Whole numbers on the x-axis represent the average online learning rate (slope of scaled percent correct linear regression for each subject across 6 blocks within a day) and numbers on the x-axis represent offline learning rate (slope of the percent correct on the last trials of the N-1 day to the first trial of the Nth day). Reduced flight path deviation indicates improved performance.

No group (DLPFC stim/sham nor M1 stim/sham) exhibited statistically significant learning (i.e., reduction of flight path displacement over time), as inter-subject variability was very high for this metric (Figures 6A–D). All groups showed inconsistent positive and negative learning slopes, and though the M1 stim group had negative online learning slopes for all 4 days, none of these reached statistical significance (*p*'s>0.1). When comparing group variances for 3D autopilot displacement over experiment days, the M1 stim group showed greater variance compared with the M1 sham group during Day 1 [Chi_(1)_ = 8.46, *p* < 0.01]. The variance for the M1 stim group, however, was significantly lower than that of DLPFC stim group for 3 trials across 3 days of training [Day 1, Trial 1: *F*_(5, 9)_ = 5.1032; Day 2, Trial 4: *F*_(6, 9)_ = 6.03; Day 3 Trial 2: *F*_(6, 7)_ = 3.99; *p* < 0.05]. Interestingly, this was also true for M1 sham vs. DLPFC stim [Day 1, Trial 1: *F*_(5, 7)_ = 13.67; Day 2 Trial 4: *F*_(6, 7)_ = 4.74; *p* < 0.001]. No other variance comparisons yielded statistically significant results (Supplementary Table S2, Figure 6). While the combined learning intercept for the M1 sham group was negative (−13140 ± 13300, *p* < 0.05) this resulted from an isolated day 1/2 offline learning rate with a large standard deviation (−29011 ± 41147).

#### Vertical speed variance

The M1 stim group exhibited the lowest average values of vertical speed variance on the final day of training (4.955 ± 0.433; Supplementary Figure S2C). This is similar to the M1 sham (Supplementary Figure S2D) value of 5.051 ± 0.502 and an improvement over DLPFC groups (5.684 ± 0.690 and 5.647 ± 0.718, for stim and sham groups, respectively, but this does not reach significance under 2-way ANOVA (*p* > 0.1; Supplementary Figures S2A,B). This appears to be derived from the relatively higher online/offline learning rates in both M1 groups as compared with the DLPFC groups, though the overall rates were not statistically significant. ANCOVA, covarying the learning rates of vertical speed variance with group identity, performed on this data shows that the slopes appear identical (*p* > 0.9 vs. null hypothesis) but the initial performance (intercept) approaches significance (*p* < 0.07).

Online, offline and meta-learning rates were largely flat, and training effects were not observes within any group (*p*'s < 0.1 level (Supplementary Table S2, Supplementary Figure S2). Because vertical speed variation is primarily a motor-centric task, it may be subject to a different learning curve that was not specifically measured during this study.

#### Autopilot vertical speed deviation

M1 sham showed significant overall offline learning, with smaller deviations of vertical speed on Day 4 as compared with Day 1 (−12.21 ± 2.51, *p* < 0.05, Supplementary Figure S3D). DLPFC sham (Supplementary Figure S3B) also had a negative slope indicating reduced deviation from ideal vertical speeds, but this was not statistically significant (−69.71 ± 952.145, *p* > 0.05). However, both of these offline learning effects were washed out when combined into overall learning rates across the 4 days (M1 sham: −5.98 ± 20.26; DLPFC sham: −17.09 ± 25.80). Overall performance did not significantly change over the course of the 4 days, and initial/final performance were not significantly different across groups (*p*'s > 0.1; Supplementary Figure S3E).

Unlike tests of G-force and flight path deviation, *F*-tests do not show any significant difference for inter-subject variance during 1st trial comparisons across all groups (*p*'s > 0.1).

#### Number of control inputs

Variance between subjects for both DLPFC groups appeared larger than that of both M1 groups (Supplementary Figure S1). However, no meta, online, offline, or combined learning rates reach significance, and no significant changes were observed between groups (see Supplementary Table S2 and Supplementary Figure S1).

### fNIRS results

#### DLPFC stimulation

##### Hboxy

**Figure 7:**
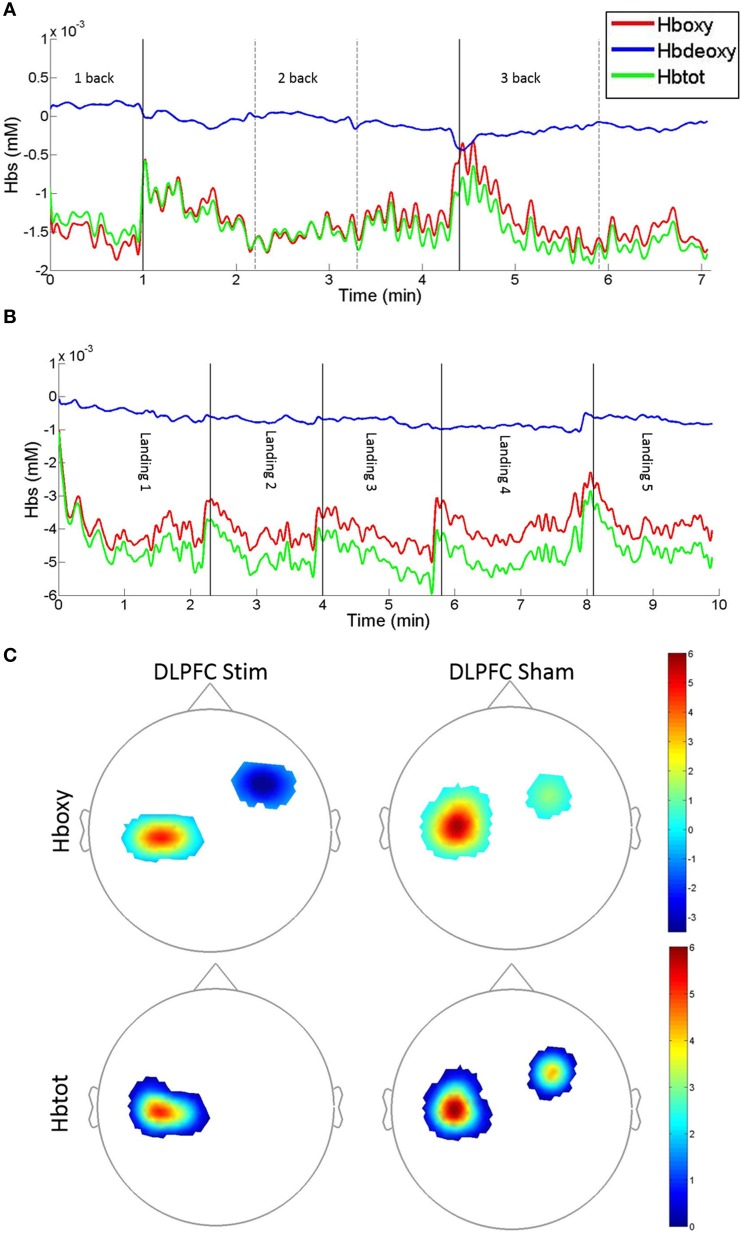
**Example fNIRS Hboxy, Hbdeoxy, and Hbtot concentration time-series and group average t-statistic beta maps. (A)** N-back concentration time series for DLPFC sham subject #S6 recorded on day 1. Traces denote DLPFC channel #10: source: FC4, detector: F4. **(B)** Easy landing blocked concentration time series for DLPFC stim subject #S1 recorded on day 1. Traces denote DLPFC channel #5: source FFC6h, detector: F4 [vertical solid and dotted lines denote blocks, solid lines indicate 1/2 and 2/3-back block types in **(A)**, and easy landing trials in **(B)**]. An upward displacement in Hboxy and Hbtot concentrations can be seen during pauses between subsequent blocks. **(C)** fNIRS t-statistic beta maps of Day 4 vs. Day 1 Hboxy (top) and Hbtot (bottom) in the Easy landing task. Images are the averages for the DLPFC stim (left) and DLPFC sham (right) groups (Bonferroni corrected *p* < 0.0025, see Table 2 for the corresponding concentration changes averaged over all channels within M1 and DLPFC).

Average Hboxy concentrations across subjects and channels significantly decreased between days 1 and 4 in M1 channels for the DLPFC stim and DLPFC sham groups, and decreased between days 1 and 4 in DLPFC channels for the DLPFC stim group (see Table 2, Figure 7).

Furthermore, individual channel analysis revealed that only two subjects (S7 in the DLPFC stim group, and S7 in the DLPFC sham group) showered a significant change in Hboxy concentrations from days 1 to 4 (−0.01 mM decrease at DLPFC channel: source AFF6h to detector F4 in DLPFC stim subject 7, and 0.02 mM increase at DLPFC channel: source FC4 to detector FFC4h in DLPFC sham subject 7, see Figure 2 for channel locations). Within the DLPFC stim group, of all 70 channels measured across subjects in the DLPFC region (10 channels per subject, 7 subjects per group) 65 showed a decrease in Hboxy concentration from days 1 to 4 (compared to 40/70, 33/60, 325/50 for DLPFC sham, M1 stim, and M1 sham respectively).

##### Hbdeoxy

The average Hbdeoxy concentration across subjects and channels within M1 significantly increased between day 1 and day 4 in the DLPFC stim and DLPFC sham groups (see Table 2). Individual channel analysis shows no significant change in Hbdeoxy concentrations from days 1 to 4.

##### Hbtot

Average Hbtot concentrations across subjects and channels significantly decreased between days 1 and 4 in M1 channels for DLPFC stim and in the DLPFC channels for the DLPFC sham group, and increased in M1 channels for the DLPFC sham group (see Table 2). Individual channel analysis revealed that only one subject (S7 in the DLPFC stim group) showed a significant change in Hbtot concentrations from days 1 to 4 (−0.01 mM decrease at DLPFC channel: source AFF6h to detector F4, see Figure 2). Within the DLPFC stim group 64/70 channels in the DLPFC region showed a decrease in Hbtot concentration from days 1 to 4 (compared to 37/70, 34/60, 32/50 for DLPFC sham, M1 stim, and M1 sham respectively).

#### M1 stimulation

##### Hboxy

Average Hboxy concentrations across subjects and channels significantly decreased between days 1 and 4 in M1 channels for the M1 stim group, and increased within M1 channels for the M1 sham group (see Table 2). Individual channel analysis shows no significant change in Hboxy concentrations from days 1 to 4.

##### Hbdeoxy

Average Hbdeoxy concentrations across subjects and channels significantly decreased between days 1 and 4 in M1 channels for the M1 stim group, and increased within M1 channels for the M1 sham group (see Table 2). Individual channel analysis shows no significant change in Hbdeoxy concentrations from days 1 to 4.

##### Hbtot

The average Hbtot concentration across subjects and channels significantly increased between days 1 and 4 in M1 channels for the M1 sham group (see Table 2). Individual channel analysis shows no significant change in Hbtot concentrations from days 1 to 4.

### EEG

#### Theta (4–7Hz)

##### DLPFC stimulation

**Figure 8:**
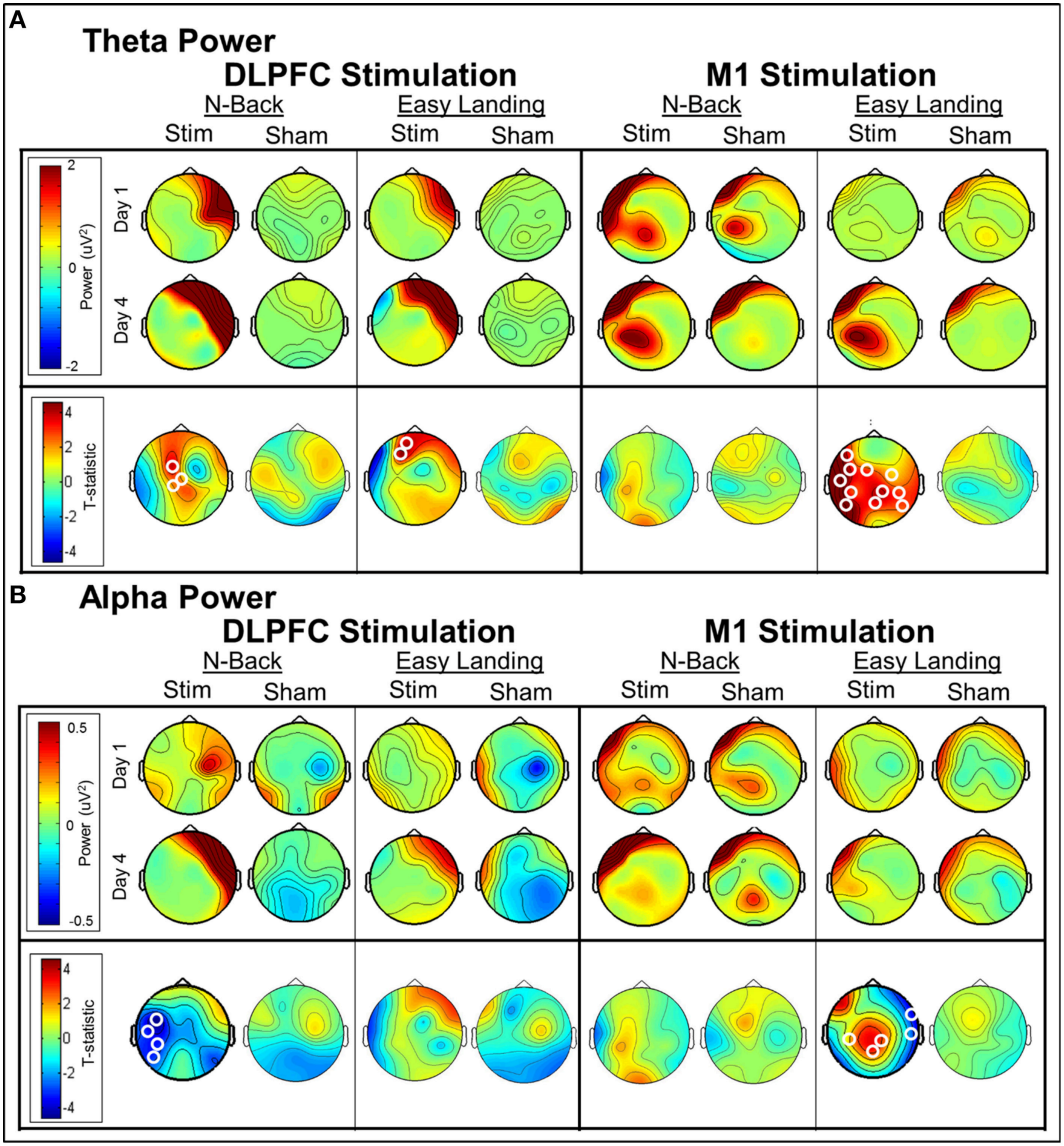
**Day 4 vs. day 1 average theta power (A) and alpha power (B) changes per group (t-statistic maps)**. Significant electrode clusters are depicted by white circles at electrode locations. Increases in midline frontal theta power were seen between days 1 and 4 in both tasks for all groups; however, cluster-level significance between days was found only for the DLPFC stim group. Decreased alpha power was found between days 1 and 4 for DLPFC stim in the N-Back task, where cluster-level significance was found in left temporoparietal electrodes. For M1 stimulation, cluster-level increases in central/parietal alpha and broadly-distributed theta power were found in the Easy Landing task.

In each day, significant differences in theta-band power were found between DLPFC stim and sham groups in frontal/central electrodes (Table 4). In days 1 and 3, right frontotemporal theta power was higher in DLPFC stim participants. Statistical differences were more broadly distributed in days 2 and 4, encompassing bilateral frontotemporal and midline frontal electrode sites. Comparison of days 1 and 4 revealed a significant increase in midline frontal theta-band power in DLPFC stim, but not DLPFC sham participants (see Figure 8A and Table 4). Split-plot ANOVA comparing MFT in the easy landing task revealed a significant main effect, with DLPFC stim greater than DLPFC sham [*F*_(1, 12)_ = 4.86, *p* = 0.048]. Additionally, an interaction was found between group and day of training [*F*_(3, 36)_ = 4.54, *p* = 0.014]. Simple-effects comparisons revealed increased MFT in stim compared to sham for only day 4 [day 4: *F*_(1, 12)_ = 6.47, *p* = 0.026]. Simple-effect of day within the DLPFC stim group reached trend-level significance [*F*_(3, 16)_ = 3.15, *p* = 0.087].

**Table 4:** **Cluster statistics for comparisons of alpha- and theta-band power during the Easy Landing task**.

	**Electrodes**	t^*^	p^**^
**THETA (4–7 Hz)**			
**DLPFC stimulation** *Actual vs. Sham* Day 1 Cluster 1	E5, FT8, C4, CP6	2.58	0.024
Day 2 Cluster 1	AF3, F3, E5, FT8, FC1, FC2, E11, T7, Cz, C4, T8, E19, CP2, CP6	3.51	0.020
Day 3 Cluster 1	FT8, E11, T8, CP6	2.99	0.008
Day 4 Cluster 1	AF3, F3, FT8, FC1, FC2, E11, Cz, C4, T8, CP5, CP2, CP6, Pz, P8, Oz	3.81	0.002
*Day 4 vs. Day 1* Actual Cluster 1	AF3, F3	3.23	0.044
**M1 stimulation** *Actual vs. Sham* Day 3 Cluster 1	Cz, CP2, CP5, P7, Pz	2.72	0.030
*Day 4 vs. Day 1* Actual Cluster 1	F7, E5, FC5, FC1, T7, C4, CP5, E19, CP2, CP6, P7, Pz, P8, Oz, PO10	3.28	0.002
**ALPHA (8–12 Hz)**			
**DLPFC stimulation** *Actual vs. Sham* Day 1 Cluster 1	CP2, CP6, Pz, Oz	2.85	0.030
Day 4 Cluster 1	E5, FT8, Fz, FC2, E11, C4, T8	2.73	0.026
Cluster 2	FT8, E11, T8	3.56	0.040
Cluster 3	AF3, F3, Fz	2.94	0.048
**M1 stimulation** *Actual vs. Sham* Day 1 Cluster 1	F3, E5, Fz, FC5, FC1, FC2, C4, CP2, CP5, CP6	2.85	0.014
*Day 4 vs. Day 1* Actual Cluster 1	CP5, E19, Pz	2.89	0.034
Cluster 2	E11, T8	−2.62	0.050

* *Reported t-values are the average t-statistic across all electrodes in a given cluster*.

** *Reported p-values are corrected for multiple comparisons using cluster-based permutation tests*.

##### M1 stimulation

Theta-band differences between M1 groups during the easy landing task were found only in day 3, and were restricted to central/parietal electrodes (Table 4). Broadly-distributed differences in theta-band power were seen between days 1 and 4 in M1 stim participants, but not M1 sham participants (see Table 4 and Figure 8A). No main effects or interactions were found in ANOVA comparing MFT in the easy landing task between M1 groups.

#### Alpha (8–12Hz)

##### DLPFC stimulation

Significant differences in alpha-band power were found between DLPFC stim and sham groups in parietal/occipital electrodes (day 1) and frontal/central electrodes (day 3), with greater power in the DLPFC stim group (Table 4). No differences in alpha power were found in the comparison of day 1 and 4, for either DLPFC stim or sham groups (see Table 4 and Figure 8B).

##### M1 stimulation

Alpha-band differences between M1 groups during the easy landing task were found only in day 1, and were broadly distributed over frontal, central, and parietal electrode sites (Table 4). Two separate clusters of significant differences in alpha-band power were seen between days 1 and 4 for M1 stim participants, but not M1 sham participants (see Table 4 and Figure 8B). The first cluster revealed increased alpha power in the M1 stim group over parietal electrode sites. The second revealed decreased alpha power in right temporal electrodes.

##### EEG/fNIRS/behavioral correlations

There were positive correlations between change in MFT power (day 4 minus day 1) and both average Hbtot and average Hboxy beta values in DLPFC channels for M1 stim subjects. The direction of this correlation indicates that increased theta from days 1 to 4 is correlated with less reduction of Hboxy/Hbtot from days 1 to 4 in DLPFC fNIRS channels (Table 5). There were also strong negative correlations between change in alpha power and both average Hbtot and average Hboxy beta values in M1 channels for M1 stim subjects, indicating that increased parietal alpha power is correlated with reduced fNIRS beta values. No correlations were identified between theta/alpha power and fNIRS beta values for sham groups, there was no correlation between theta and fNIRS beta values at M1 channels, and there was no correlation between alpha power and fNIRS beta values at DLPFC channels (*p*'s > 0.1).

**Table 5:** **Significant correlations among EEG, fNIRS, and behavioral results in the Easy Landing task, for the M1 stim group**.

	***r***	***p***
**SIGNIFICANT EEG/BEHAVIOR CORRELATIONS**		
*Day 2* *Online Learning* MFT × Number of control inputs	0.66	0.038
MFT × autopilot displacement	0.85	0.002
MFT × VSDFA	0.84	0.002
MFT × vertical speed variance	0.84	0.002
*Offline learning* MFT × autopilot displacement	−0.85	0.002
MFT × VSDFA	−0.85	0.002
MFT × vertical speed variance	−0.85	0.002
*Day 4* *Online Learning* Broadly-Distributed Theta Power × VSDFA	−0.70	0.025
Central/Parietal Alpha Power × VSDFA	−0.85	0.002
		
**SIGNIFICANT EEG/fNIRS CORRELATIONS (DAY 4–DAY 1)**		
MFT × Hbtot	0.82	0.045
MFT × Hboxy	0.81	0.051
Parietal Alpha Power × Hbtot	−0.95	0.005
Parietal Alpha Power × Hboxy	−0.94	0.005

*VSDFA, Vertical Speed Deviance from Autopilot*.

## Discussion

### Overview

In this study, we measured task-evoked changes in functional neural activity and the modulation of learning from tDCS to the right DLPFC or left M1. Simultaneous fNIRS and EEG measured changes in neural activity as subjects learned to complete flight simulator and n-back training exercises at increasing levels of expertise across four daily consecutive sessions. Assessment of behavioral performances were performed on n-back accuracy, flight metrics of landing performance, as well as for online and offline learning rates associated with practice and skill acquisition. We report that tDCS to the right DLPFC reduced the variability in online learning across individuals in the n-back task, and in g-force on the easy landing task. This was associated with decreased Hboxy and Hbtot in the DLFPC across days for the landing task, and increased MFT power in both the n-back and landing tasks. Additionally, tDCS to the left M1 increased tonic parietal alpha power, which was correlated with changes in Hboxy and Hbtot at M1 fNIRS channels.

### Interpretation—behavior

The observed reduction in group variability in online learning may be attributed to “convergence to the mean” (i.e., increasing online learning rates of low performing individuals and reducing online learning rates of high performing individuals). Subjects may have employed distinct cognitive and behavioral strategies, with correspondingly different brain networks, to complete and learn the n-back task across sessions. tDCS of the right DLPFC may have therefore facilitated the deployment and consolidation of a particular strategy in some subjects, and inhibited certain behaviors in others. The variance in the learning rates did not arise from individual differences of untrained performance, as initial and final performances were similar (see Section Behavioral Results). Furthermore, the results could indicate that all groups reached a ceiling of behavioral performance, or that our measures are under-powered to detect a change in performance statistically, or that a reduction in individual variability produced this observation.

The variability results reported for the easy-landing task were specific to DLPFC stim subjects for the g-force metric [a similar reduction in variance was not seen for the same data in the autopilot displacement (Figure 6), the number of control inputs (Supplementary Figure S1), the variability of vertical speed (Supplementary Figure S2), and the vertical speed deviation from autopilot (Supplementary Figure S3)]. Since both the initial and final g-force values were not significantly different across stim and sham groups, the reduction in DLPFC stim group variability implies a similar convergence to the mean phenomenon observed for n-back learning. tDCS of the DLPFC may therefore, facilitate the learning of a smoother landing procedure in subjects who would otherwise consolidated an incorrect landing procedure and increased landing g-forces in subsequent days. Likewise, tDCS of the DLPFC may have hindered some subjects who would have otherwise consolidated a superior landing procedure and decreased landing g-forces in subsequent days.

It should be noted that for the measure of 3D autopilot flight path deviation (Section 3D Autopilot Displacement), it was not readily apparent to subjects when the aircraft deviated from the prescribed flight path of the autopilot; there is no visual field indication that they are deviating from the glide slope, and the Flight Director instrument does not indicate degree of displacement from optimal glide slope. Additionally, for deviation from the autopilots vertical speed (Section Autopilot Vertical Speed Deviation) is possible that, because vertical speed was a peripheral skill required for landing (i.e., non-essential for a successful landing), subjects did not train to maintain a low vertical speed deviation from the reference glide path. As subjects needed only to maintain one constant vertical speed during the landing task, they may have reached maximal capacity to do so beginning from day 1. The combined learning rates and online learning metrics seem to support this view (see Supplementary Table S2, Supplementary Figure S3). Furthermore, low-G Force landings can be performed from a wide range of glide slopes, which can mask large deviations from the “ideal” flight path.

### Interpretation—neurophysiology

We observed an increase in MFT in the DLPFC stim group compared to the DLPFC sham group, as well as experience-related increase in MFT and decrease in central/parietal alpha in DLPFC stim, indicating increased working memory and attention (Klimesch et al., 1997; Jensen and Tesche, 2002; Ishii et al., 2014). Increased theta/alpha band activity in M1 stim compared to M1 sham near the site of stimulation may indicate greater motor cortex excitability (Sauseng et al., 2009). Furthermore, experience-related increases (day 4 vs. 1) in broad central/parietal theta/alpha in M1 stim during flight tasks implicate greater tactile/proprioceptive monitoring. For example, Baumeister et al. (2008) observed that increased parietal theta during goal-directed learning was associated with increased motor skill performance. Although MFT nor parietal alpha power increases were correlated with behavioral performance increases in this study, it is possible that increases in MFT or parietal alpha may be indirectly associated with cognitive performance enhancement. The significant correlations observed between MFT and online and offline learning of autopilot displacement, vertical speed variance and deviation in the M1 stim group support this hypothesis (Table 5).

We observed a decrease in Hboxy and Hbtot in DLPFC channels for the DLPFC stim group in the easy landing task (Table 2 and results Hboxy and Hbtot). This evidence suggests that tDCS produced more efficient neural activation to consolidate the newly-learned procedural skills as has been previously reported (Wolf et al., 2007; Holland et al., 2011; Ayaz et al., 2012; DiStasio and Francis, 2013). Previous literature from McKendrick et al. (2015) suggest that some, but not all, of these changes may be related to the task performance enhancements associated with tDCS. However, changes in Hbtot concentration may also be related to task reward value (DiStasio and Francis, 2013), the recruitment of additional motor resources (Herff et al., 2013), or a behavioral ceiling effect where low-performing subjects were not able to advance to expert performance levels as shown by Ayaz et al. (2012). Although reward was not explicitly manipulated in the easy landing task, subject's motivations may have played a role based on their prior day's performance. Similarly, the motor resources required for the easy landing task may have changed as subjects learned more advanced motor programs to complete the task. Finally, a ceiling effect could explain the more efficient neural activation, and the trend in meta-learning for stim groups supports this theory (Figure 5E).

Hbtot and Hboxy were also significantly correlated with MFT from days 4 to 1 in the easy landing task in the M1 stim group. These results suggest that these separate neurophysiological measures are not totally independent. Future studies should examine the relationships between MFT, Hbtot, and behavioral performance in a larger cohort to determine whether these effects are truly concomitant.

### Relation to prior investigations of tDCS in real-world tasks

To date, there have been few studies in which procedural/real world learning tasks have been tested with a tDCS intervention (Izzetoglu et al., 2014; Nelson et al., 2014), and even fewer with a significant motor component as the focus of performance/training enhancement (Zhu et al., 2015). However, tDCS enhancement of real-world skills has been reported for complex motor control tasks. For example, Beeli et al. (2008) reported that anodal tDCS to either the left or right DLPFC (10/20 EEG site F3 or F4) significantly improved the care of driving style as measures by following distance, average speed and number of errors. Similarly, Sakai et al. (2014) reported that anodal tDCS to the right DLPFC significantly improved car-following and lane-keeping performance in a driving simulator task across days. Finally, Zhu et al. (2015) reported that cathodal tDCS to the left DLPFC suppressed verbal working memory but improved motor learning. The results presented here support these findings, as we observed that tDCS to the right DLPFC reduced online learning variability in higher cognitive measures (e.g., affecting the g-force value of landing by judging multi-modal flight-data in a timely fashion, or n-back accuracy variance) more than those related motor planning or judgment (e.g., flight path deviation see Table 6).

**Table 6:** **Summary of behavioral and neurophysiological results**.

**Group**	**N-Back**	**G-force**	**fNIRS**	**EEG**
DLPFC Stim	- Variance	- Variance	- Hboxy and Hbtot in the DLPFC (flight only)	+ MFT power (N-back and flight)
M1 Stim				+ Parietal Alpha power (flight only)

Furthermore, real-world skill enhancement from right inferior frontal tDCS has been reported in a perceptual threat detection (Clark et al., 2012; Falcone et al., 2012), and tDCS of the DLPFC has been shown to increase regional cerebral blood oxygenation and behavioral performance in target detection in an air traffic control task (Nelson et al., 2014). The results presented here are indirectly related to these findings as the reduction in behavioral variance we observed from tDCS to the right DLPFC could be attributed to increase in spatial attention, vigilance, or perceptual discrimination (e.g., when to judge an n-back match or the correct time for a nose-flare maneuver during landing). We also observed that tDCS of the right DLPFC decreased Hboxy and Hbtot in DLFPC channels across days in the easy landing task. One possible explanation for the difference reported in cerebral blood oxygenation between the two studies concerns the disparate experimental designs employed. Here, all subjects returned for four consecutive days of testing, regardless of physiological or behavioral measures, whereas Nelson et al. (2014) had subjects return for days 2–4 only if performance and blood flow velocity declined over the course of the first 40-min session.

### Relation to prior investigations of tDCS in working memory

Previous studies have reported evidence that working memory improvements are correlated with the administration of tDCS in diverse contexts (Grafman et al., 1994; Nitsche et al., 2003; Dockery et al., 2009; McKendrick et al., 2015). Specifically, tDCS over DLPFC was associated with acute increases in working memory accuracy (Stagg and Johansen-Berg, 2013; Chhatbar and Feng, 2015; De Putter et al., 2015; Santarnecchi et al., 2015). Although, we observed a reduction in learning rate variance from tDCS to the right DLPFC in the n-back task, but did not find an increase in working memory accuracy for tDCS of either the DLPFC or M1. This discrepancy may be attributed to the adaptive n-back design employed here, the long durations of experimental sessions, and a potential ceiling effect from repeated tDCS and n-back sessions across consecutive days. In addition, the application of tDCS began directly prior to the n-back task (see Figure 1) and the effects of stimulation may require more time to produce the reported improvements in n-back accuracy.

### Limitations and future directions

A goal of this research was to determine if tDCS stimulation would improve training techniques for pilots in a flight simulator. Such improvements could drastically reduce time and therefore the cost of training a pilot, as it would in any training environment. While our results show decreased variability in training, it is too early to confirm or deny any useful improvements to simulation training until an understanding of the sources and contributing factors to the observed behavioral variance is achieved.

Additional studies must be performed to further investigate n-back accuracy improvement with tDCS by comparing different stimulation montages, stimulation timing, and task paradigms. Because we were unable to parametrically manipulate these parameters in this study, we are unable to determine which of these factors may have led to null effects of tDCS on n-back accuracy. The baseline performance of individuals with differing initial skill levels in n-back and flight tasks are important, and measures of this were limited by the study design employed. In addition, the experimental design employed here (continuous, multiple tasks over 60 min duration) did not provide a sufficient means to control the endogenous brain state of subjects before and throughout the experimental session given the numerous tasks, and instructions and feedback required for subjects to perform them. Thus, subject's diverse experiences and resultant brain states throughout the session may be a significant factor in the interpretation of our findings. For example, the n-back task was performed near the beginning of the stimulation period, while the easy flight landing was performed near the end of the stimulation period. Future studies should examine relationships between tDCS effects and EEG microstates and/or brain metabolic activity.

Some of the null findings in this study were related to exceptionally high within-group variance. One potential method to examine within and across group behavioral variance is to categorize subjects by learning rate bins or perform a cluster analysis of tDCS responders and non-responders. Since the same tDCS protocol may have variable effects across individuals, possibly due to neuroanatomical and neurophysiological differences, and that the same tDCS protocol may produce different effects within an individual over time, due to changes resulting from neural plasticity, the absence of *post-hoc* categorization of subjects likely reduces the statistical power and interpretability of our results (e.g., Supplementary Table S2). Future studies may benefit from real-time assessments and individualized tDCS planning rather than a “one size fits all” approach. While a priori selection or *post-hoc* classification of subjects within experimental groups can control for differences in baseline performance levels, it is not realistic when transferring this technology into real-world training environments.

The high variability between subjects and the need for personalized training becomes more important when we recognize the subject pool for this experiment all fit the western, educated, industrialized, rich and democratic (WEIRD) population. Although this population of subjects for the experiment goal of pilot training was acceptable, we speculate that the inclusion of a wider demographic range of the world populous may produce an even larger variability in behavioral performance. Therefore, a systematic understanding of the sources and contributing factors to the observed behavioral variance is extremely important for the application of tDCS across a wider range of subjects.

## Conclusions

The results presented here underscore the importance of developing the understanding to identify and optimize neurostimulation protocols. Our results suggest that the time course of both online and offline learning is critical for the observed changes in working memory and procedural flight performance. Repeated training sessions reveal time-dependent factors regarding the interaction between tDCS and the learning processes that remain unclear in the literature. Applying such interventions in the real-world will require a much larger investment than initially anticipated in order for the scientific community to measure and catalog the precise behavioral, learning, and neurophysiological changes resulting from each component of procedural skill acquisition. Because there appears to be a differential, region-based effect of neurostimulation interventions, it is critical to determine the optimal targets, stimulation parameters, timing relative to the target behaviors, and synchrony between innate learning processes and strategies and exogenous stimulation for maximally-effective augmentation.

## Author contributions

JC, MZ, and MP designed the experiments; JC and DB performed the experiments; JC, BC, and MP analyzed the data; and JC, BC, and MP wrote the manuscript.

### Conflict of interest statement

The authors declare that the research was conducted in the absence of any commercial or financial relationships that could be construed as a potential conflict of interest.
