# Supplementary material for: Transcranial Direct Current Stimulation Modulates Neuronal Activity and Learning in Pilot Training
Source: Front Hum Neurosci. 2016 Feb 9;10:34. doi: 10.3389/fnhum.2016.00034 (PMC4746294; doi:10.3389/fnhum.2016.00034)
Supplement: Supplementary file 1 [file Image1.PDF]

Supplementary Figure S1. Major control input results across all four experimental groups. A-D) Average number of major control inputs to aircraft during easy landing is shown for each group. Variance between subjects is very high for all experimental groups, though the environmental constraints remained identical throughout all trials. E) Online and offline control input learning rates are plotted for each experimental group across the duration of the experiment. Whole numbers on the x-axis represent the average online learning rate (slope of scaled percent correct linear regression for each subject across 6 blocks within a day) and ½ numbers on the x-axis represent offline learning rate (slope of the percent correct on the last trials of the N-1 day to the first trial of the Nth day) . For control inputs, DLPFC stim seems to increase the number of inputs throughout the training period compared to sham.

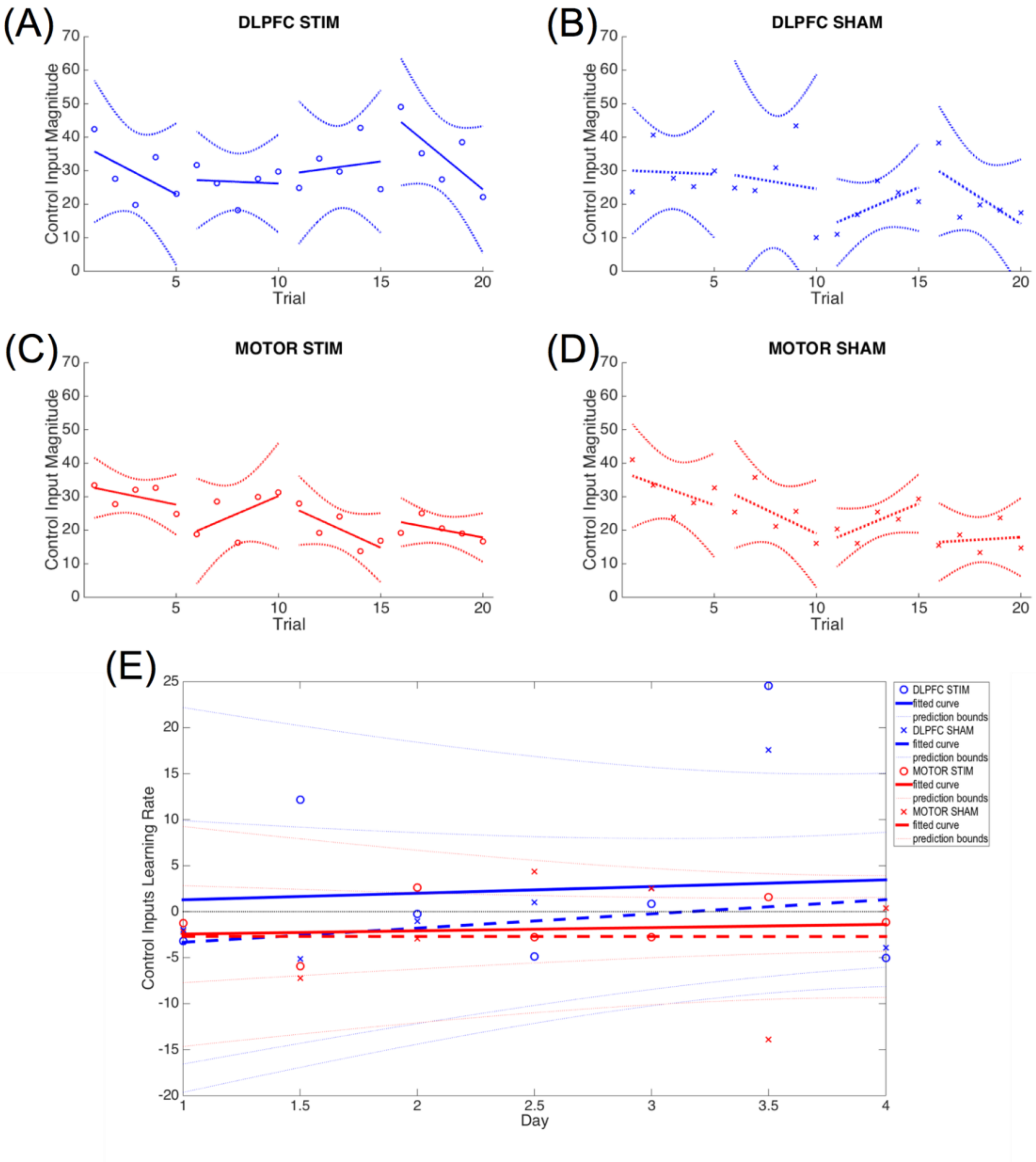

Supplementary Figure S2. Vertical Speed variance results across all four experimental groups. A-D)Average vertical Speed variance during easy landing is shown for each group. Learning appears inconsistent during online periods, though some changes in variance (confidence intervals) point to inter-subject consistency changes depending upon stimulation condition. E) Online and offline vertical speed variance learning rates are plotted for each experimental group across the duration of the experiment. Whole numbers on the x-axis represent the average online learning rate (slope of scaled percent correct linear regression for each subject across 6 blocks within a day) and ½ numbers on the x-axis represent offline learning rate (slope of the percent correct on the last trials of the N-1 day to the first trial of the Nth day). For vertical speed deviation, there appears to be no learning trend for all any subject group.

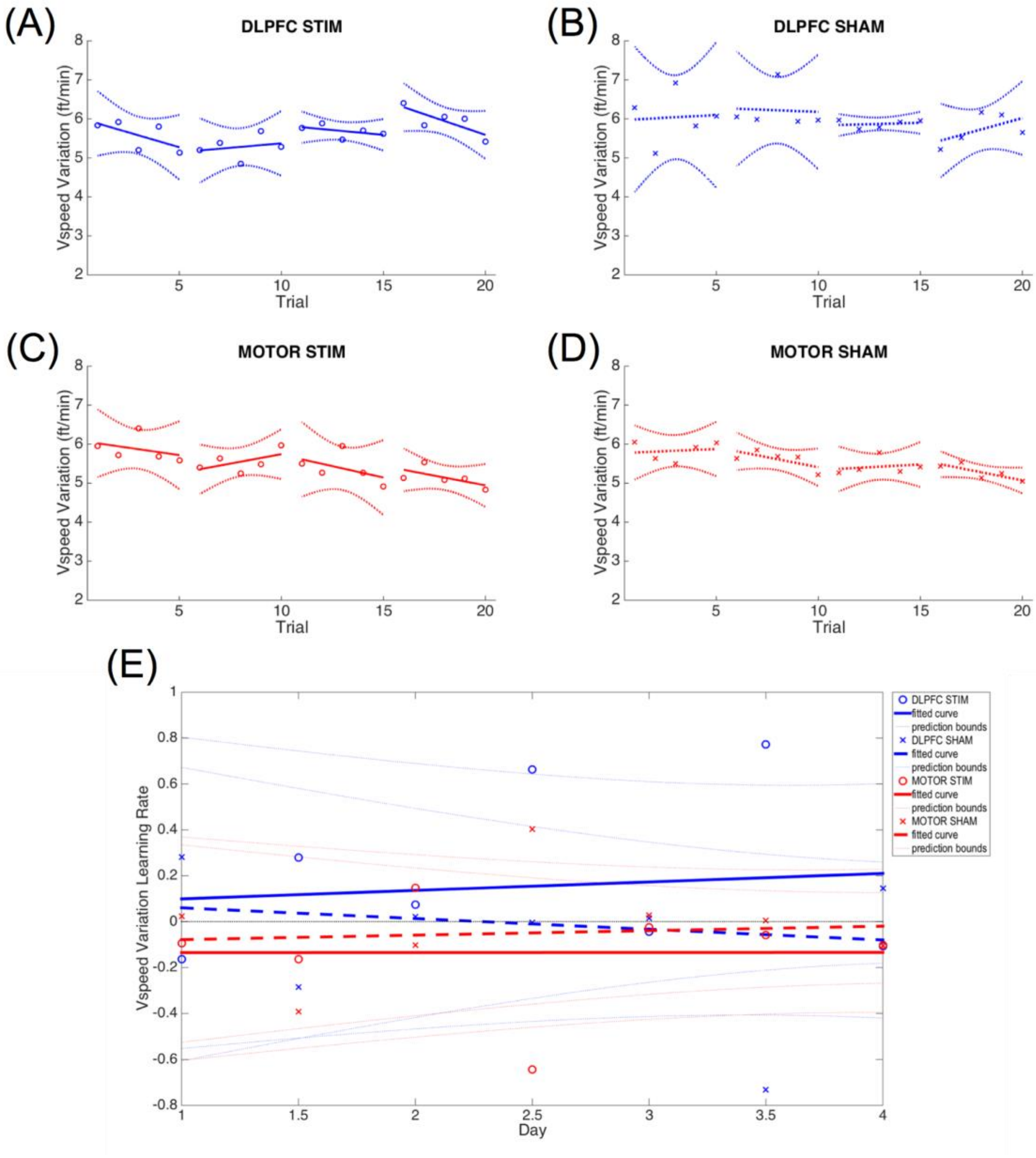

Supplemental Figure S3. Vertical Speed deviation results across all four experimental groups. A-D) Average vertical Speed deviation from autopilot reference slope during easy landing is shown for each group. Learning appears inconsistent during online periods, though some changes in variance (confidence intervals) point to inter-subject consistency changes depending upon stimulation condition. E) Online and offline vertical speed deviation learning rates are plotted for each experimental group across the duration of the experiment. Whole numbers on the x-axis represent the average online learning rate (slope of scaled percent correct linear regression for each subject across 6 blocks within a day) and ½ numbers on the x-axis represent offline learning rate (slope of the percent correct on the last trials of the N-1 day to the first trial of the Nth day) . For vertical speed deviation, there appears to be no learning trend for any subject group. Smaller deviation from the reference vertical speed indicates improved performance.

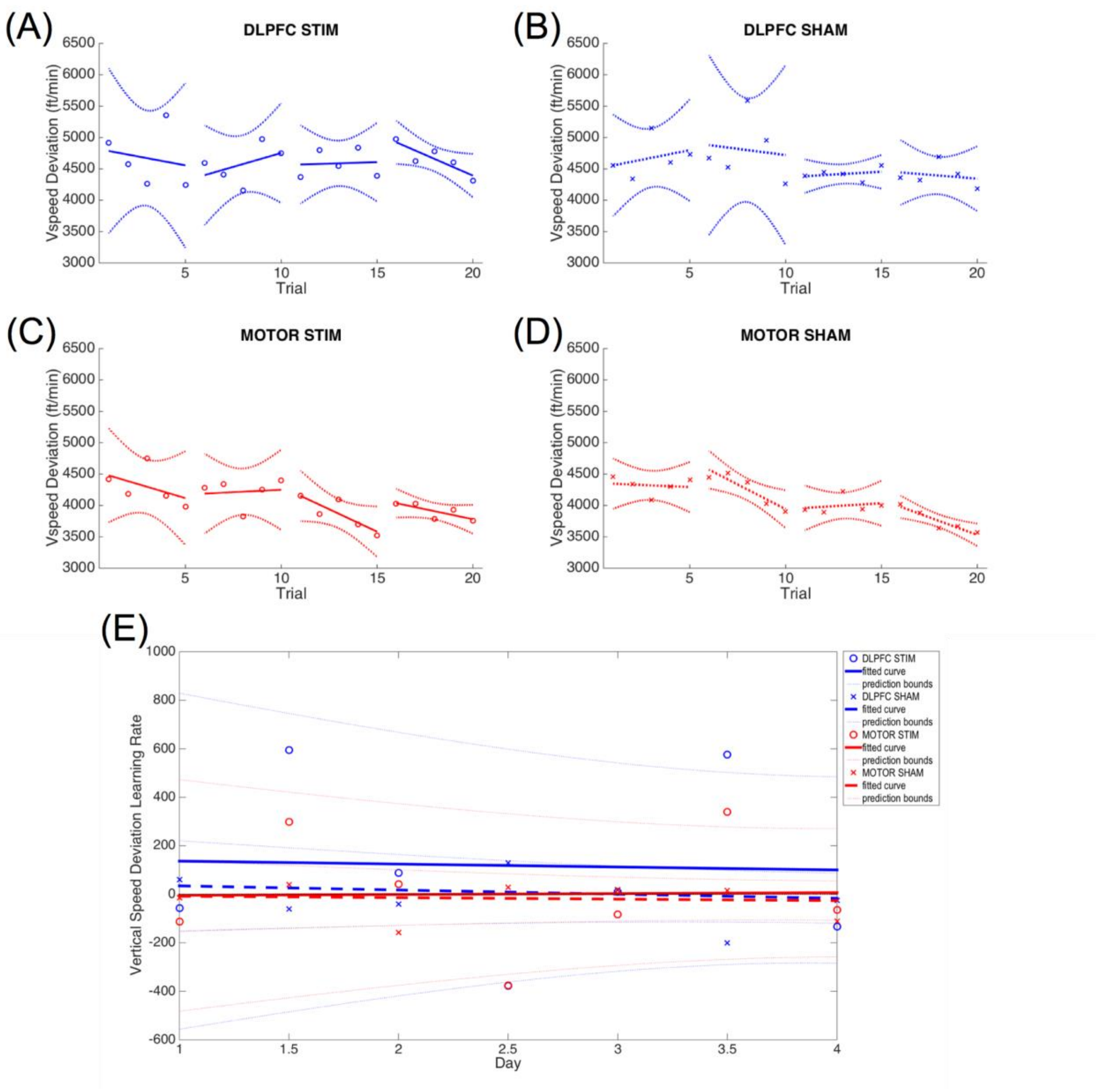

Supplementary Figure S4. Baseline-subtracted alpha power (A) and beta power (B) during the finger-tapping task, across all subjects. Sensorimotor network activity is seen as an increase in beta power and a decrease in alpha power, compared to baseline.

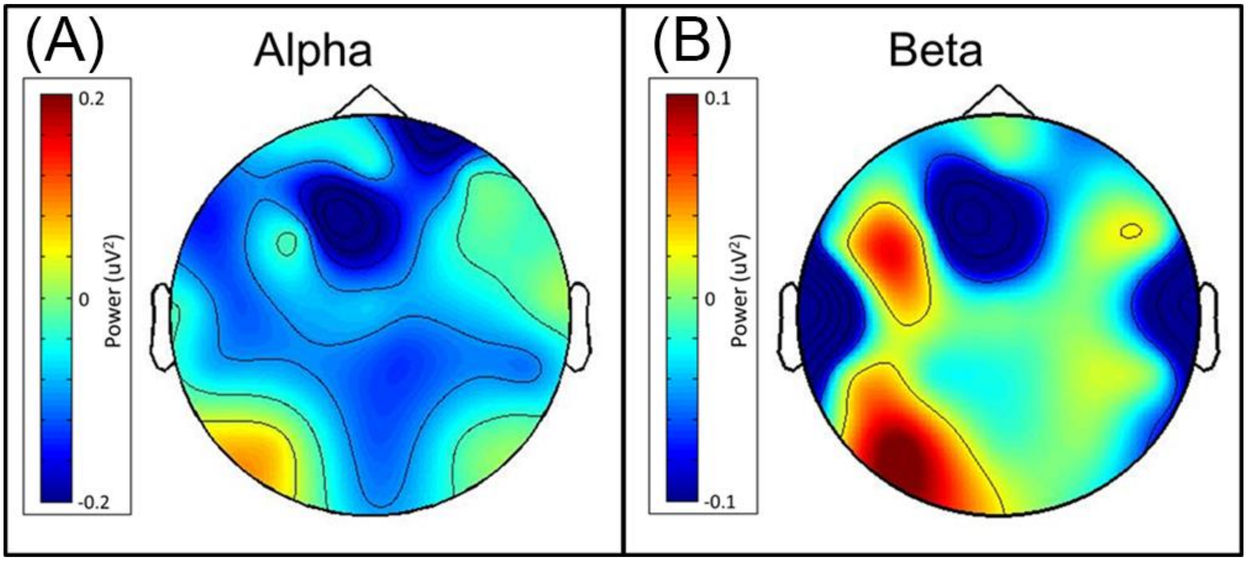

Supplementary Figure S5. fNIRS t-statistic beta map of finger tapping task-evoked activity compared to baseline. Image shows the group average Hbdeoxy activation across all 32 subjects during day 1. SPM t-stat map image ($p<0.05$) of Hbdeoxy (Hbtot and Hboxy showed no significant group-level activation at this threshold).

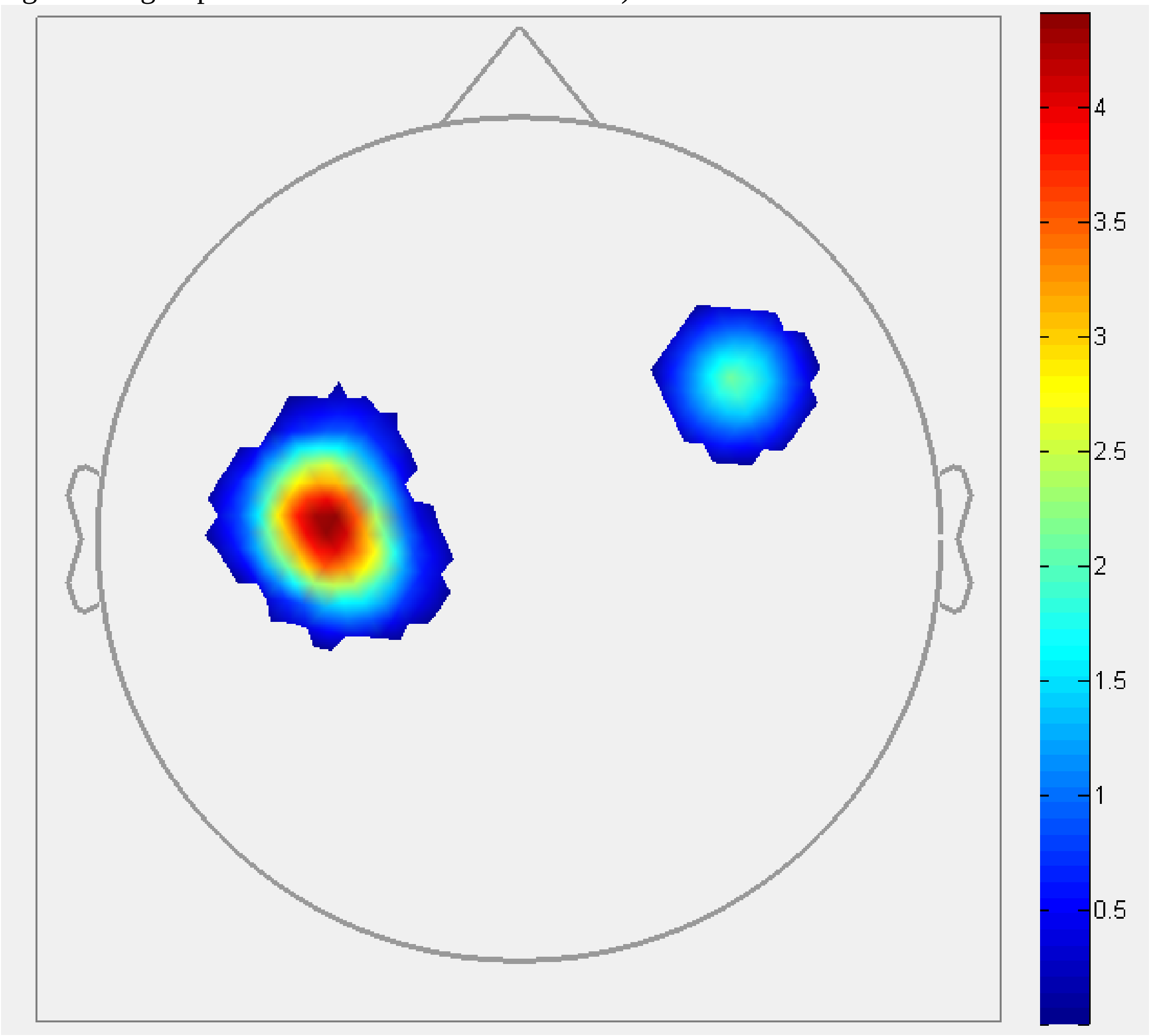

Supplemental Table S1. Summary of N-back results significant results are denoted in bold text (one-tailed t-test versus zero)

**Group**	Initial 1-back % correct (scaled) (Combined, Position, Image)	Avg. % correct across all (scaled)/1/2/3 Backs (not scaled) (Combined, Position, Image)	Final % correct (last trial last day, scaled) (Combined, Position, Image)	Average trials to reach 2/3 Back	Avg. trial streak @ 1/2/3 back	Avg. % of trials @ 1/2/3 back	Learning rate over Avg. group accuracy for all (scaled)/1/2/3 Backs (not scaled) (Combined, Position, Image)	Avg. *online* learning rate for initial (1st day)/Avg./Final(4th day) (Combined, Position, Image)	Avg. *offline* learning rate for initial (1st day)/Avg./Final(4th day) (Combined, Position, Image)	Avg. *combined* learning rate (across all online and offline rates) (Combined, Position, Image)
**dlPFC stim**	C: 13.6±6.0<br>P: 16.0±10.8<br>I: 12.5±6.7	C: 21.2±10.1/57.9±24.6/36.4±18.2/26.0±7.1<br>P: 23.6±15.9/56.3±33.1/45.7±29.1/43.5±9.2<br>I: 19.7±10.9/60.7±28.9/29.5±19.1/12.5±17.7	C: 28.0±4.0<br>P: 28.4±8.4<br>I: 25.4±7.5	10.1±6.6<br>23	4.2±6.2<br>3.2±1.0<br>2 (1 e.g.)	60±22%<br>39±20%<br>1±3%	C: **0.52±0.11/1.56±0.36**/0.53±0.39/10<br>P: **0.87±0.17/1.64±0.50**/1.09±0.62/-13<br>I: 0.23±0.12/**1.55±0.43**/0.00±0.41/25	C: 0.44±1.39/0.84±1.74/0.44±2.70<br>P: -0.01±3.69/0.41±2.73/-0.40±3.49<br>I: 1.72±1.66/1.41±2.16/0.96±3.12	C: 1.70±5.50/3.12±11.56/-0.94±14.88<br>P: 2.03±10.29/0.19±10.60/-3.91±13.11<br>I: 4.62±11.06/6.58±15.87/1.38±19.68	C: 1.85±3.20<br>P: 0.27±2.07<br>I: 3.68±4.76
**dlPFC sham**	C: 18.5±8.3<br>P: 17.7±10.1<br>I: 18.4±9.9	C: 28.3±15.7/56.7±25.6/47.8±20.9/52.1±16.6<br>P: 34.8±24.8/61.2±29.5/57.3±34.2/80.4±16.7<br>I: 22.1±14.4/53.0±30.9/39.5±23.5/21.7±18.1	C: 46.8±28.0<br>P: 53.4±35.7<br>I: 38.7±20.7	5±3.3<br>5	3.6±7.3<br>5.1±3.2<br>5 (1 e.g.)	40±35%<br>51±32%<br>9±24%	C:**0.61±0.18**/0.58±0.47/0.18±0.38/1.61<br>P: **1.04±0.28**/0.75±0.55/0.77±0.62/1.72<br>I: 0.20±0.17/0.43±0.58/-0.23±0.43/1.08	C: 1.44±2.16/0.92±2.39/2.91±2.19<br>P: 3.86±4.82/1.52±4.13/0.92±3.38<br>I: 0.03±3.15/0.78±3.65/4.91±5.16	C: -3.58±9.14/-2.39±14.53/6.72±16.80<br>P: 3.38±12.55/-0.38±19.53/2.71±27.05<br>I: -10.80±12.82/-3.30±14.66/7.99±17.34	C: -0.18±4.88<br>P: 0.67±3.87<br>I: -0.38±6.05
**Motor stim**	C: 14.9±7.5<br>P: 17.6±11.2<br>I: 11.9±10.0	C: 26.1±11.9/63.2±24.6/45.8±19.0/30.8±14.5<br>P: 31.4±18.8/64.1±31.3/58.4±28.5/45.9±27.3<br>I: 20.4±12.5/63.0±29.5/32.2±21.1/11.4±14.2	C: 29.1±10.4<br>P: 33.0±21.4<br>I: 25.7±17.5	7.3±5.9<br>18.8±7.2	3.8±2.9<br>4.3±2.0<br>2.9±0.7	44±29%<br>50±22%<br>7±11%	C: **0.37±0.11/0.68±0.33**/0.45±0.28/-1.05<br>P: **0.45±0.17**/0.31±0.43/0.27±0.43/-2.20<br>I: **0.24±0.11/0.95±0.40**/0.53±0.31/0.68	C: 1.55±1.82/0.88±2.35/-0.10±3.35<br>P: 2.18±3.81/0.73±4.06/-1.18±5.78<br>I: 0.79±3.33/0.81±2.93/0.66±3.15	C: 0.99±12.63/1.24±12.15/6.70±12.52<br>P: -1.88±16.73/-0.40±16.71/4.37±19.78<br>I: 2.08±19.67/2.51±17.55/6.68±16.40	C: 1.03±3.13<br>P: 0.25±2.72<br>I: 1.54±2.52
**Motor sham**	C: 15.2±6.5<br>P: 15.4±11.7<br>I: 16.1±6.4	C: 29.5±12.1/73.3±21.7/47.6±19.8/31.6±12.9<br>P: 35.8±18.8/73.2±29.5/59.8±28.5/43.5±22.8<br>I: 23.9±13.7/69.9±25.0/37.2±23.7/22.8±13.8	C: 35.5±11.3<br>P: 51.9±18.9<br>I: 20.4±13.2	4.6±1.7<br>16.3±7.2	1.7±1.1<br>3.8±1.1<br>2.3±1.0	27±11%<br>60±5%<br>13±15%	C: **0.69±0.12/2.26±0.38/0.92±0.30**/0.34<br>P: **1.17±0.18**/1.26±0.66/**1.74±0.42**/0.51<br>I: 0.28±0.14/**2.15±0.49**/0.35±0.38/0.00	C: 1.41±2.10/0.83±2.14/1.71±1.76<br>P: 2.31±2.57/1.67±3.12/2.66±4.38<br>I: 0.53±2.66/-0.29±3.14/-0.02±2.85	C: -6.75±12.3/-3.08±16.21/-5.54±16.59<br>P: -4.33±13.28/-0.65±18.24/-2.61±16.85<br>I: -8.99±23.01/-6.16±23.98/-12.95±25.66	C: -0.87±3.73<br>P: 0.66±3.14<br>I: -2.92±5.73

Supplemental Table S2. Means and standard deviations for flight metrics. Online learning indicates learning slopes derived from within-day subject performance across trials. Offline learning indicates between-day performance. Combined learning is the slope of interleaved offline and online learning slopes. For all tasks, minimization of behavioral metrics indicates improved performance. As such, negative values for learning rate (slope) indicate skill improvement in the task. No values for all stimulation/shame groups were found to be statistically significant.

			**Online Learning**		
	**Day 1**	**Day 2**	**Day 3**	**Day 4**	**Trend**
**DLPFC STIM**					
G-force	0.13 ± 0.22	-0.55 ± 0.18	-0.090 ± 0.12	-0.16 ± 0.23	-0.067 ± 0.10
Position Deviation	-5457.9 ± 15052	2024.2 ± 10988	-2026.6 ± 6824.7	-4646.1 ± 13444	-865.7 ± 5746.3
V-speed Deviation	-57.32 ± 258.63	88.34 ± 350.13	9.16 ± 252.06	-132.89 ± 157.86	-30.59 ± 100.91
V-speed Variation	-0.16 ± 0.30	0.07 ± 0.17	-0.04 ± 0.25	-0.11 ± 0.26	0.00 ± 0.12
Control Inputs	-3.18 ± 4.90	-0.26 ± 8.95	0.83 ± 6.90	-5.03 ± 10	-0.45 ± 3.09
**DLPFC SHAM**					
G-force	0.078 ± 0.20	-0.00 ± 0.30	-0.07 ± 0.21	-0.04 ± 0.22	-0.05 ± 0.11
Position Deviation	-1400.3 ± 14618	-3537.5 ± 10639	239.8 ± 3903.7	4575.9 ± 9648	2170.6 ± 4108.7
V-speed Deviation	60.97 ± 357.65	-39.68 ± 205.87	17.73 ± 266.46	-24.75 ± 139.38	-19.97 ± 44.00
V-speed Variation	0.28 ± 0.73	0.02 ± 0.20	0.01 ± 0.45	0.15 ± 0.14	0.04 ± 0.06
Control Input	-1.99 ± 8.09	-1.03 ± 4.13	2.60 ± 4.40	-3.94 ± 10.19	-0.22 ± 3.21
**MOTOR STIM**					
G-force	0.01 ± 0.50	0.066 ± 0.42	-0.11 ± 0.16	-0.03 ± 0.14	-0.03 ± 0.12
Position Deviation	-524.2 ± 6607.9	-1098 ± 6273	-4555.2 ± 8554.8	-1962.3 ± 11848	-1030.1 ± 4470.2
V-speed Deviation	-113.22 ± 187.80	42.10 ± 374.36	-82.70 ± 229.71	-64.07 ± 87.85	1.27 ± 81.33
V-speed Variance	-0.09 ± 0.30	0.15 ± 0.54	-0.03 ± 0.25	-0.10 ± 0.20	-0.02 ± 0.13
Control Inputs	-1.25 ± 7.43	2.61 ± 6.56	-2.78 ± 6.09	-1.14 ± 4.42	-0.51 ± 2.59
**MOTOR SHAM**					
G-force	-0.00 ± 0.26	-0.16 ± 0.21	-0.06 ± 0.09	-0.08 ± 0.18	-0.014 ± 0.12
Position Deviation	1887.9 ± 9437.6	-170.2 ± 8071.9	-3004.3 ± 4469.8	156.3 ± 8604.8	-802.8 ± 4410.8
V-speed Deviation	-13.51 ± 314.05	-157.73 ± 250.75	18.47 ± 172.07	-111.74 ± 126.97	-11.85 ± 95.48
V-speed Variation	0.02 ± 0.37	-0.10 ± 0.35	0.03 ± 0.19	-0.10 ± 0.17	-0.03 ± 0.08
Control Inputs	-2.19 ± 11.56	-2.91 ± 6.73	2.54 ± 6.013	0.36 ± 3.62	1.31 ± 2.16
		**Offline Learning**			**Combined Learning**
	**Day 1/2**	**Day 2/3**	**Day 3/4**	**Trend**	**Trend**
**DLPFC STIM**					
G-force	-0.15 ± 0.53	0.08 ± 0.84	0.64 ±1.05	0.22 ± 0.70	0.052 ± 0.09
Position Deviation	45.9 ± 45203	-8274.8 ± 27572	27605 ± 38151	25210 ± 42967	1911 ± 1114.5
V-speed Deviation	594.19 ± 1137.2	-376.89 ± 1252.8	576.670 ± 884.665	-8.75 ± 3529.6	-12.17 ± 96.065
V-speed Variation	0.28 ± 0.18	0.66 ± 0.81	0.77 ± 1.19	0.25 ± 0.51	0.04 ± 0.10
Control Inputs	12.17 ± 32.74	-4.86 ± 41.18	24.57 ± 42.02	6.20 ± 85.20	0.73 ± 2.90
**DLPFC SHAM**					
G-force	0.12 ± 0.99	0.72 ± 0.90	-0.08 ± 0.89	-0.26 ± 2.55	-0.05 ± 0.11
Position Deviation	5818.8 ± 77582	12052 ± 17930	-11241 ± 27499	-8530.1 ± 31120	-443.3 ± 1971.3
V-speed Deviation	-60.19 ± 1471.4	129.73 ± 266.20	-199.58 ± 1018.9	-69.70 ± 952.15	-17.09 ± 25.80
V-speed Variation	-0.29 ± 2.99	-0.00 ± 1.48	-0.73 ± 1.24	0.35 ± 1.38	-0.05 ± 0.85
Control Input	-5.14 ± 23.31	1.00 ± 11.80	17.57±45.88	11.36 ± 19.13	1.54 ± 1.83
**MOTOR STIM**					
G-force	-0.24 ± 0.68	-0.03 ± 0.95	0.42 ± 0.84	0.13 ± 0.49	0.04 ± 0.05
Position Deviation	5471 ± 33718	8091.2 ± 20308	9566 ± 32315	809.2 ± 30178	-1056 ± 1935.5
V-speed Deviation	299.54 ± 1269.3	-375.19 ± 1639.4	340.60 ± 433.41	20.52 ± 2550.3	3.74 ± 66.17
V-speed Variance	-0.16 ± 1.25	-0.64 ± 2.79	-0.058 ± 0.95	0.052 ± 1.95	0.00 ± 0.065
Control Inputs	-5.90 ± 16.748	-2.78 ± 26.56	1.56 ± 19.30	3.73 ± 2.22	0.35 ± 0.73
**MOTOR SHAM**					
G-force	0.26 ± 1.33	-0.02 ± 0.51	0.22 ± 0.49	0.84 ± 0.78	-0.08 ± 0.41
Position Deviation	-29011 ± 41147	-105.9 ± 20290	11734 ± 22550	20373 ± 24113	2624 ± 2975.5
V-speed Deviation	39.67 ± 1327	28.15 ± 459.7	15.26 ± 1006	-12.21 ± 2.505	-5.98 ± 20.26
V-speed Variation	-0.39 ± 1.51	0.40 ± 0.52	0.01 ± 1.22	0.20 ± 0.86	0.02 ± 0.06
Control Inputs	-7.25 ± 36.15	4.38 ± 11.14	-13.88 ± 32.15	-3.31 ± 54.79	-0.01 ± 1.66
